# Design and model choices shape inference of age-varying genetic effects on complex traits

**DOI:** 10.1038/s43587-026-01232-w

**Published:** 2026-10-06

**Authors:** Tabea Schoeler, Simon Wiegrebe, Thomas W. Winkler, Zoltán Kutalik

**Affiliations:** 1 Department of Computational Biology, University of Lausanne, Lausanne, Switzerland; 2 Department of Clinical, Educational and Health Psychology, University College London, London, UK; 3 Swiss Institute of Bioinformatics, Lausanne, Switzerland; 4 University Center for Primary Care and Public Health, Lausanne, Switzerland; 5 Department of Genetic Epidemiology, University of Regensburg, Regensburg, Germany; 6 Statistical Consulting Unit StaBLab, Department of Statistics, LMU Munich, Munich, Germany

**Keywords:** Genetic interaction, Genetic association study, Ageing

## Abstract

Understanding how genetic influences on complex traits change with age is a fundamental question in genetic epidemiology. Both cross-sectional (between-subject) and longitudinal (within-subject) approaches can contribute to answering this question but come with distinct strengths and limitations. Here we show that age-varying genetic effects obtained from the two designs were highly concordant in direction (84.21% of the 57 identified variants) but showed only moderate agreement in effect-size magnitude (Pearsonʼs r= 0.51). Confounding by gene-by-birth year effects accounted for the largest proportion of variance in effect-size differences across single-nucleotide polymorphisms (SNPs) with age-varying effects between designs (70.8%). Participation bias accounted for an additional 11.6%, whereas unmodeled nonlinear age trajectories contributed minimally to these differences (4.2%). Overall, our results demonstrate that both cross-sectional and longitudinal designs can yield different estimates of age-varying genetic effects, principally due to cohort confounding and participation bias. As neither approach is immune to design-specific limitations, we recommend integrating both designs for robust inference, to help improve interpretability and more accurately characterize how genetic effects on complex traits change over the lifespan.

## Abstract (web summary)

Genetic influences on complex traits change over the lifespan. Schoeler et al. show that cross-sectional and longitudinal studies can give different estimates of how genetic effects change with age, mainly owing to birth cohort differences and participation bias.

## Main

A central question in lifecourse and developmental genetic epidemiology is whether, and how, the impact of genetic variation on complex traits changes with age. Understanding age-varying genetic effects provides critical insights into developmental processes, differential aging trajectories and the etiology of age-related diseases. Explicit modeling of genome-wide age-varying effects can, thus, reveal important patterns of diminishing (or intensifying) genetic influence across the lifecourse patterns that would be missed by standard genome-wide association tests screening for average effects across time.

Detecting and characterizing age-varying genetic effects is crucial not only for elucidating underlying biological mechanisms but also for ensuring robust inference in genetic epidemiology. For example, age-dependent effects may obscure SNP heritability estimates, contributing to the so-called ‘missing heritability’^1–3^. Furthermore, such varying effects create challenges for an array of methods assuming stable genetic effects across different age groups, including Mendelian randomization (MR)^4^, fixed-effects genome-wide meta-analyses^5,6^ or polygenic prediction^7^. Given the documented evidence of varying genetic effects across the lifespan^8,9^, such presence can, therefore, complicate the interpretation of common genomic outputs.

**Fig. 1:**
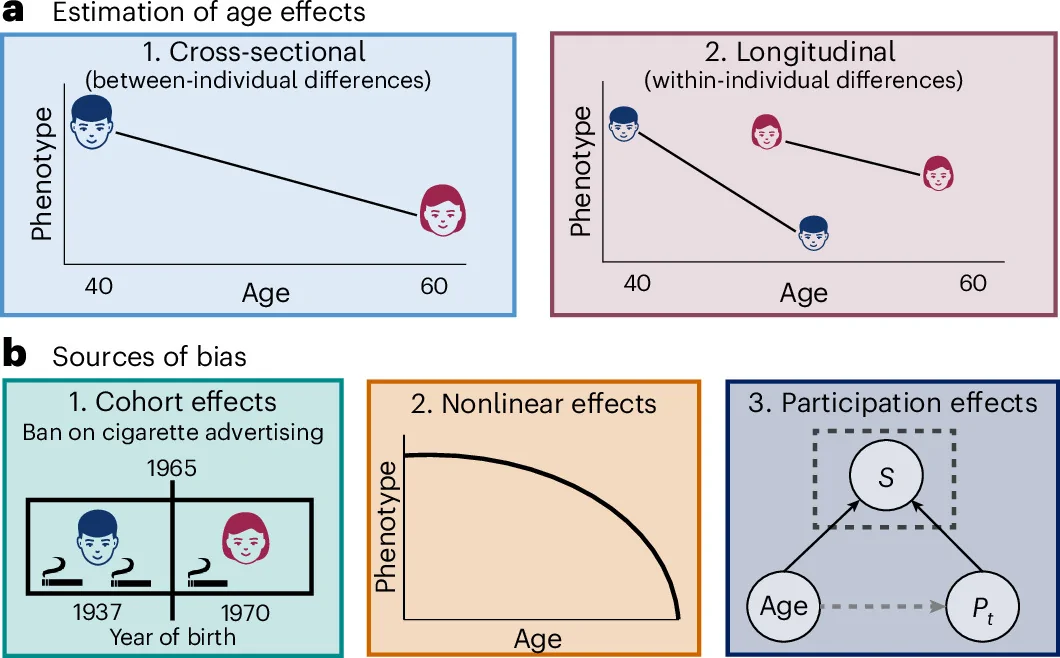
Study framework. **a**, Cross-sectional (between-individual, in blue) and longitudinal (within-individual, in red) design used for the identification and estimation of age and age-varying genetic effects. **b**, Possible sources of bias when estimating age and age-varying genetic effects, including (1) birth cohort effects (differences arising from historical or societal changes affecting people born at different times—for example, the 1965 UK ban on cigarette advertising reducing cigarette use in younger cohorts); (2) nonlinear age effects (where age effects depend on the age of the individual); and (3) selective participation (where selection into the sample, *S*, depends on age and the phenotype, *P*, assessed at timepoint *t*).

Traditionally, investigations into age-dependent genetic effects have predominantly utilized cross-sectional gene-by-age interaction tests, indirectly approximating age-related changes by comparing genetic associations across individuals of differing ages^10–14^ (Fig. 1a, panel 1). By contrast, longitudinal assessments provide a more direct measure of the aging process, by modeling within-individual change over time and its genetic determinants (Fig. 1a, panel 2). Although longitudinal genome-wide analyses were previously constrained by the lack of repeated measures in large cohorts, the emergence of large-scale prospective biobanks such as the UK Biobank (UKB) now enables the estimation of genetic effects on change at genome-wide scale^15–18^.

Both cross-sectional and longitudinal approaches have been used to study age-varying genetic effects and related downstream processes^10–14,18–21^, often implicitly assuming that they target the same quantity of interest (that is, the same estimand). However, agreement between the two designs requires strong assumptions, including the absence of cohort- or period-by-genotype effect modification, alongside comparable sampling and study participation characteristics. For example, cross-sectional designs often benefit from significantly larger samples and wider age ranges (for example, 40−69 years in approximately 500,000 UKB participants), thereby maximizing statistical power and reducing false negatives^19^. Additionally, they are potentially less susceptible to selective participation (Fig. 1b, panel 3) than longitudinal designs, in which (age-related) participation bias may be amplified due to selective dropout or survival. Conversely, longitudinal designs, despite smaller sample sizes and shorter follow-up windows (for example, approximately 10 years in up to 100,000 UKB participants), provide more robust control against time-invariant sources of confounding, particularly gene-by-birth year (that is, cohort) interactions (Fig. 1b, panel 1). Cohort effects reflect differences attributable to the historical and social contexts in which individuals were born and raised, such as shifts in educational systems, economic conditions or public policies. Age effects, by contrast, refer to changes arising from the aging process itself as individuals grow older, such as declines in physiological function or cognitive performance. Because age and year of birth are inherently linked in cross-sectional data (age = period − cohort), between-cohort differences can be misattributed to aging if not explicitly accounted for. For example, cohort-varying genetic effects have been documented in response to societal changes, such as the collapse of the Soviet Union^22,23^ or educational policy reforms^24^—effects that would falsely be interpreted as age-varying genetic effects if tested cross-sectionally. Additionally, longitudinal data with multiple repeated measures enable the detection of nonlinear age-related changes, offering deeper insights into age-varying genetic effects that deviate from linear trends (Fig. 1b, panel 2).

Given the distinct limitations and strengths characterizing cross-sectional and longitudinal designs, it is critical to understand their relative impact on genome-wide findings and inference. In this work, we, therefore, systematically evaluate age-varying genetic effects estimated from both approaches, identify sources of discrepancy and provide guidance on their optimal application in genome-wide analyses on complex traits.

## Results

### Cross-sectional versus longitudinal age effects

**Fig. 2:**
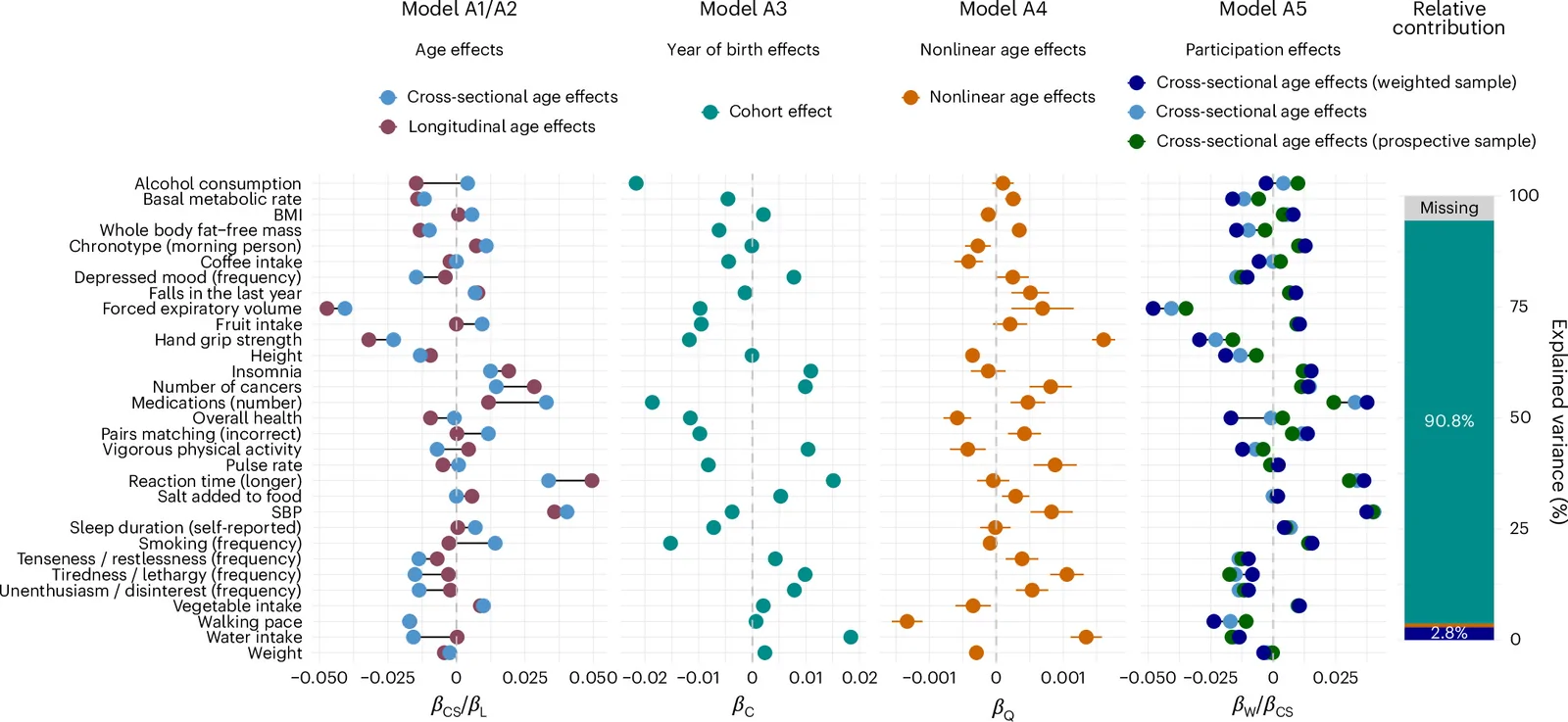
Cross-sectional and longitudinal age effects. The plot for models A1/A2 shows the age effects obtained from cross-sectional analyses (${\beta }_{\mathrm{CS}}$ in model A1, highlighted in light blue) and longitudinal analyses (${\beta }_{\mathrm{L}}$ in model A2, highlighted in red), where negative coefficients indicate that older age links to decline in trait values. The plot for model A3 shows the birth year effects obtained from model A3 (${\beta }_{\mathrm{C}}$, in cyan), where negative coefficients indicate that later birth year links to lower trait values. The plot for model A4 shows the nonlinear age effects (${\beta }_{\mathrm{Q}}$ in model A4, in orange). The plot for model A5 contrasts the baseline participation adjusted cross-sectional age effects (${\beta }_{\mathrm{W}}$, obtained from model A5, in dark blue) to cross-sectional age effects obtained from the UKB baseline sample (${\beta }_{\mathrm{CS}}$ in model A1, highlighted in light blue) and the longitudinal UKB sample (in green). The error bars represent the 95% confidence intervals. The bar plot to the right indicates how much of the variance (in %) in effect-size differences between longitudinal and cross-sectional age effects (${\beta }_{L}-{\beta }_{{CS}}$) was explained by cohort effects (in cyan), nonlinear age effects (in orange) and selective participation (in dark blue). The source data used in this figure can be found in Supplementary Data 1.

To evaluate the convergence between cross-sectional and longitudinal age effects, we focused our analysis on 31 non-binary health-related phenotypes in the UKB, spanning domains relevant to cognition, physiological traits, clinical indicators, anthropometric measures and lifestyle and behavioral factors. Figure 2 presents the age effects obtained from the cross-sectional model (A1, up to N= 498,845 individuals) and the longitudinal model (A2, up to N= 99,459 with at least one follow-up assessment). A summary of all model estimates and corresponding samples sizes is available in Supplementary Data 1. Overall, cross-sectional age effects explained 70.57% of the variance in longitudinal estimates. The largest difference in age effects was present for medications (number), where the cross-sectional model substantially overestimated the within-person age effect, with ${\beta }_{\mathrm{CS}}=$ 0.03 ($\mathrm{s.e.}=$ 0.0002) and ${\beta }_{\mathrm{L}}=$ 0.01 ($\mathrm{s.e.}=$ 0.0002), due to confounding via cohort effects (further discussed below). Inconsistencies in direction of effects (that is, opposite sign) were detected for seven of the 31 included traits, particularly among lifestyle-related traits (for example, alcohol use, smoking, coffee intake and physical activity).

To better understand the factors contributing to discrepancies between cross-sectional and longitudinal estimates, we examined three possible contributors, including birth cohort effects (model A3, up to N= 99,459), nonlinear age effects (model A4, up to N= 19,374) and selective participation (model A5, up to ${N}_{\mathrm{eff}}=$ 141,147). Significant Bonferroni-corrected cohort effects (discovery threshold: P<0.05/31) were detected for 28 of the 31 assessed traits (Fig. 2, model A3). Because date of birth and cross-sectional age are highly inversely correlated in the UKB (r=−0.99), negative cohort effects systematically inflate cross-sectional age estimates and, in cases of strong cohort confounding, may even reverse the direction of the observed age effect. For example, substantial negative cohort effects for smoking and alcohol use explained why these traits, which decline with age when assessed longitudinally, falsely appeared to increase with age when tested in cross-sectional analyses. Overall, cohort effects accounted for 90.8% of the variance in discrepancies between longitudinal and cross-sectional estimates (Fig. 2), thus representing the primary driver of effect discrepancies between the two designs.

Significant quadratic age effects were detected for 17 traits (${\beta }_{\mathrm{Q}}\ne 0$ at $P < 0.05\,/\,31$; Fig. 2, model A4), indicating that aging trajectories deviated from linearity for some phenotypes. In such cases, models assuming linearity, whether cross-sectional or longitudinal, produce biased estimates, either underestimating or overestimating age effects at the extremes of the age spectrum. Because the age ranges differ between the cross-sectional and longitudinal UKB samples (for example, a maximum baseline age of 69 years versus 86 years at follow-up), the estimated age slopes may differ when nonlinear effects are present (see Supplementary Fig. 1 for illustration). However, the relative importance of quadratic age effects appeared to be negligible, accounting for 0.9% (P= 0.015) of the variance in effect size differences between designs.

Finally, we assessed the influence of sample representativeness by comparing the cross-sectional age effects across three subsamples: a baseline sample adjusted for selective participation (most representative), the unweighted baseline sample and the longitudinal follow-up sample (least representative). Traits for which the UKB sample was the least representative produced more attenuated cross-sectional age effect estimates, with sign reversals observed for four traits (Fig. 2, model A5). Although sample representativeness significantly predicted discrepancies between cross-sectional and longitudinal estimates, its contribution was only small compared to the variation attributable to birth year effects (explained variance: 2.8% versus 90.8%, respectively). Because selective dropout may influence longitudinal age estimates, we also derived inverse probability weights for participation in follow-up assessments (Supplementary Information). As shown in Supplementary Fig. 2, reweighting the follow-up sample to improve representativeness had, overall, a smaller impact on longitudinal age estimates than the impact observed after reweighting of the cross-sectional sample to account for selective baseline participation. Of note, of the 399,661 participants not taking part in follow-up research, 42,001 (11%) died prior to the first longitudinal assessment. Because the majority of loss to follow-up was due to reasons other than death, the observed changes in effect estimates likely reflect selective dropout mechanisms weakly related to mortality.

### Cross-sectional versus longitudinal age-varying genetic effects

**Fig. 3:**
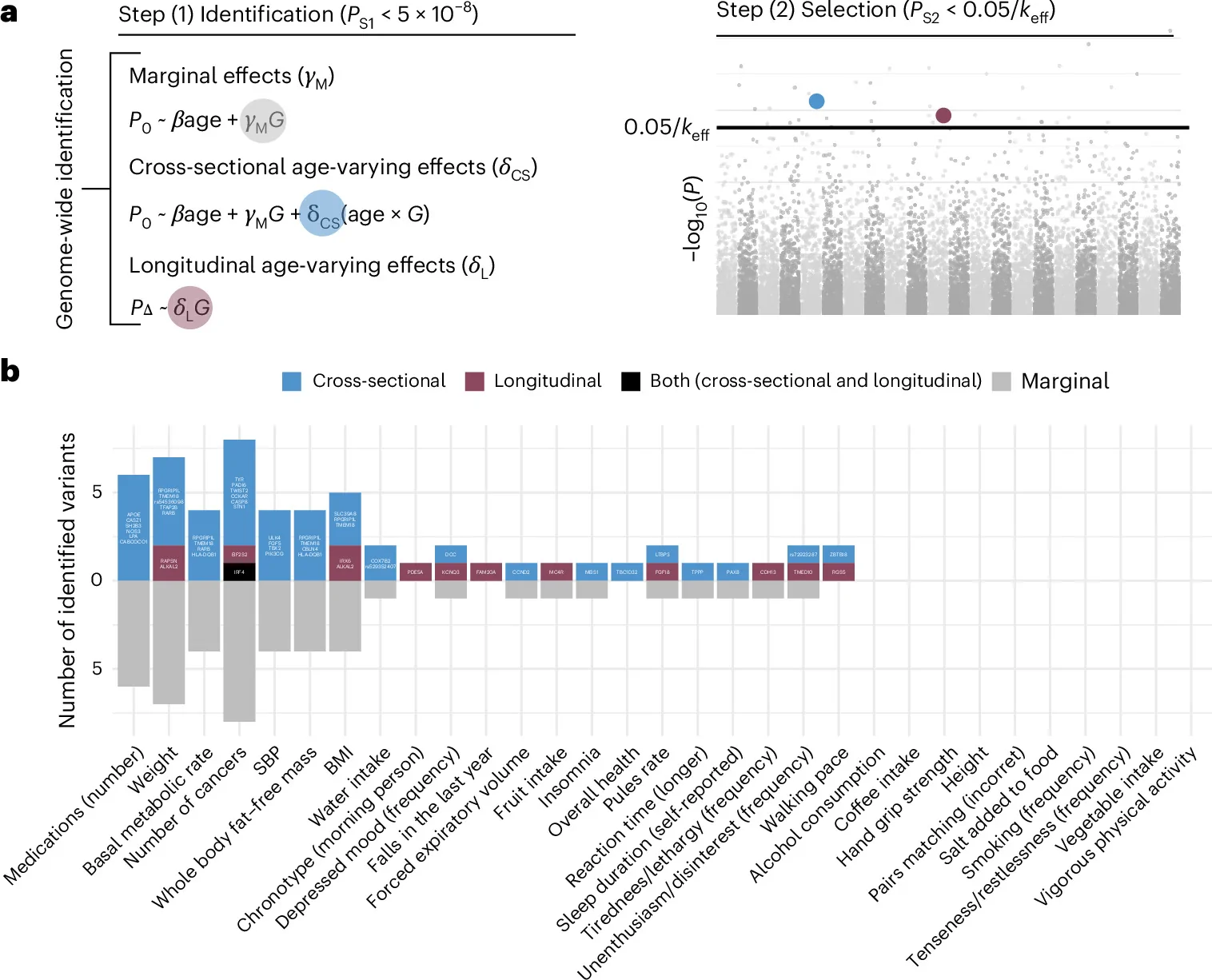
Cross-sectional and longitudinal identification of age-varying genetic effects. **a**, Two-step genome-wide approach combining marginal (in gray), cross-sectional (in light blue) and longitudinal (in red) models to detect age-varying genetic effects. In step (1), we performed genome-wide scans for models on marginal genetic effects (${\gamma }_{M}$), cross-sectional gene-by-age interaction effects (${\delta }_{\mathrm{CS}}$) and genetic effects on change (${\delta }_{\mathrm{L}}$). In these equations, ~ denotes ‘is modelled as a function of.’ In step (2), we selected all LD-independent SNPs reaching genome-wide significance ($P < 5\times {10}^{-8}$) in any of the three models as candidate genes. We then selected all Bonferroni-corrected significant variants (discovery threshold: ${P}_{S2} < 0.05/{k}_{\mathrm{eff}}$) from the two models testing for age-varying genetic effects (${\delta }_{\mathrm{CS}}$, ${\delta }_{\mathrm{L}}$), where ${k}_{\mathrm{eff}}$ corresponds to the total number of LD-independent variants reaching genome-wide significance per trait identified in step 1. Annual age-related change (${{\mathrm{P}}}_{{\Delta }}$) is derived based on the difference between the standardized phenotype assessed at baseline (${P}_{0}$) and the most recent follow-up assessment (${P}_{1}$), divided by the follow-up time. G is the genetic variant. The estimated age-varying genetic effects (δ) can be interpreted as the genetic effect on annual change. **b**, All Bonferroni-corrected significant (${P}_{\mathrm{S}2} < 0.05\,/\,{{\mathrm{k}}}_{\mathrm{eff}}$) genetic variants (mapped to closest gene) identified in cross-sectional gene-by-age interaction tests (in light blue), in longitudinal tests of age-varying genetic effects (in red) or in both sets of analyses (black). The gray bars at the bottom indicate the number of Bonferroni-corrected significant variants with indicated age-varying genetic effects that were also significant in tests of marginal effects. The source data used in **b** can be found in Supplementary Data 2. LD, linkage disequilibrium.

We identified 57 linkage-disequilibrium-independent, Bonferroni-corrected variants with significant age-varying effects. Most of these were detected in cross-sectional analyses (${k}_{\mathrm{SNPs}}=$ 44, up to 406,226 individuals), with fewer reaching significance in longitudinal analyses (${k}_{\mathrm{SNPs}}=$ 14, up to 83,579 individuals). Most of the identified variants were linked to anthropometric traits (weight, body fat−free mass, body mass index (BMI)), metabolic indicators (basal metabolic rate) and clinical outcomes (number of medications used, number of cancers) (Fig. 3b). By contrast, self-reported health behaviors (for example, physical activity, smoking and sleep) exhibited no or only few significant age-varying genetic effects. The source data used in Fig. 3b are provided in Supplementary Data 2.

**Fig. 4:**
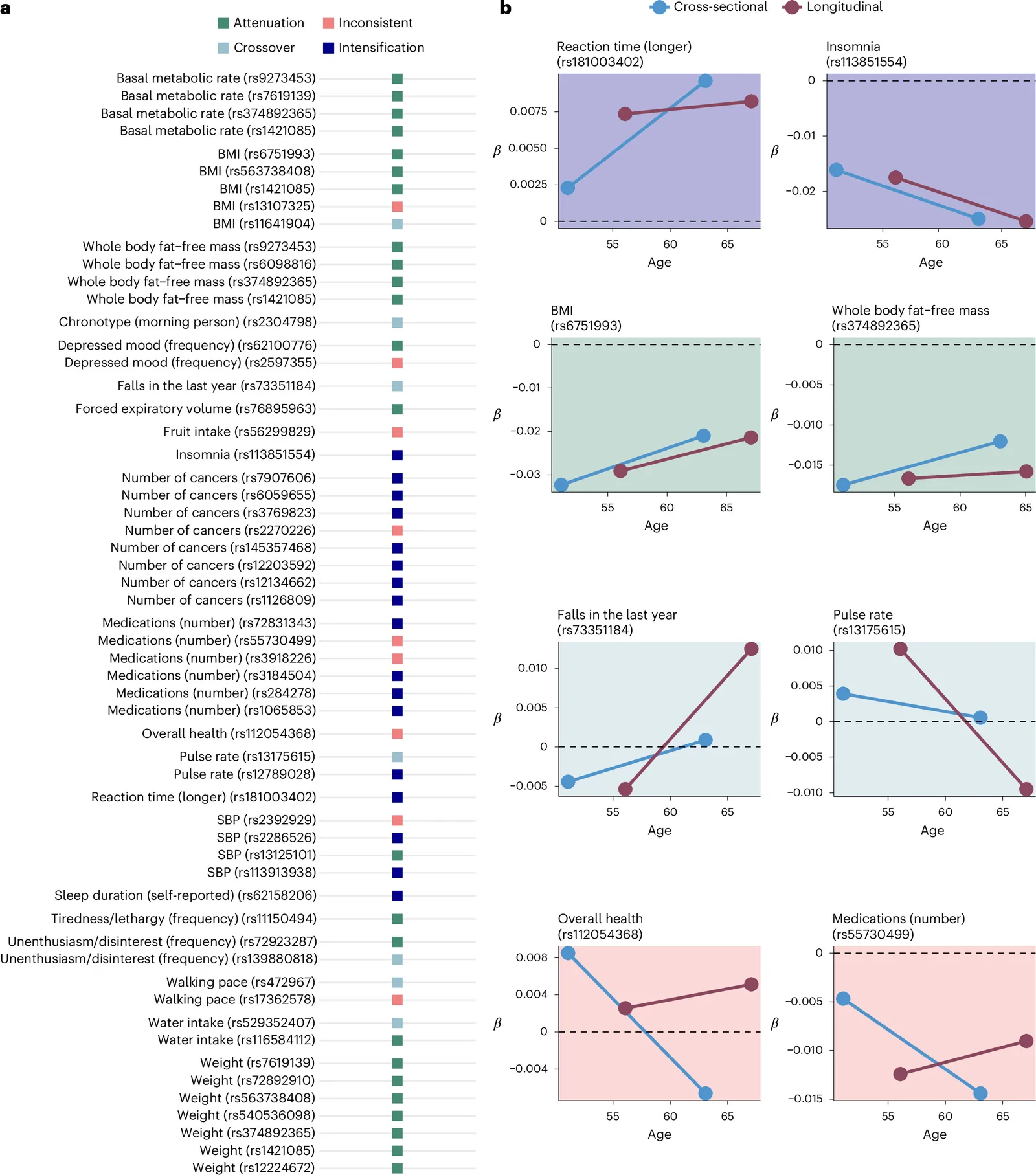
Cross-sectional and longitudinal interpretations of age-varying genetic effects. **a**, All Bonferroni-corrected significant variants identified in the cross-sectional and/or longitudinal analysis. The color scheme refers to the interpretation of the age-varying genetic effects, which was inferred based on the direction of the main genetic effect (${\gamma }_{M}$) and the age-varying genetic effect (δ) for a given variant, inferring attenuation (main and age-varying effect have opposite signs, in light green), intensification (main and age-varying effect have the same direction, in blue) or crossover (significant age-varying effect are present without a significant marginal effect, in light blue) of genetic effects over time. Variants highlighted in coral are those with inconsistent direction of age-varying genetic effects (that is, opposite sign) obtained from the cross-sectional and longitudinal approach. Panel **b** visually illustrates the mechanim of interaction, by plotting the main genetic effect (in standardized coefficients) obtained in younger versus older age groups selected from the baseline UKB sample (splitting individuals around the median age, in light blue) or the prospective UKB sample (using the baseline and follow-up assessment, in red). The *x* axis indexes the mean age of the sample used to estimate the genetic effects.

Regarding the direction of effects, most of the 57 identified variants (84.21%) showed consistent interaction directions across the cross-sectional and longitudinal model. Among those variants, both attenuation and intensification were common modes of interaction (50% and 35.42%, respectively) (Fig. 4a). This pattern appeared to be trait specific: genetic effects on obesogenic traits tended to attenuate with age, whereas traits adversely impacted by aging (for example, number of cancers, medication burden and reaction time) more often showed intensifying genetic effects over time. By contrast, crossover effects were rare (14.58%), with only seven variants exhibiting age-varying effects without detectable marginal effects. In those instances, the direction of the genetic association reversed over time (see panel highlighted in blue, Fig. 4b, for illustration).

**Fig. 5:**
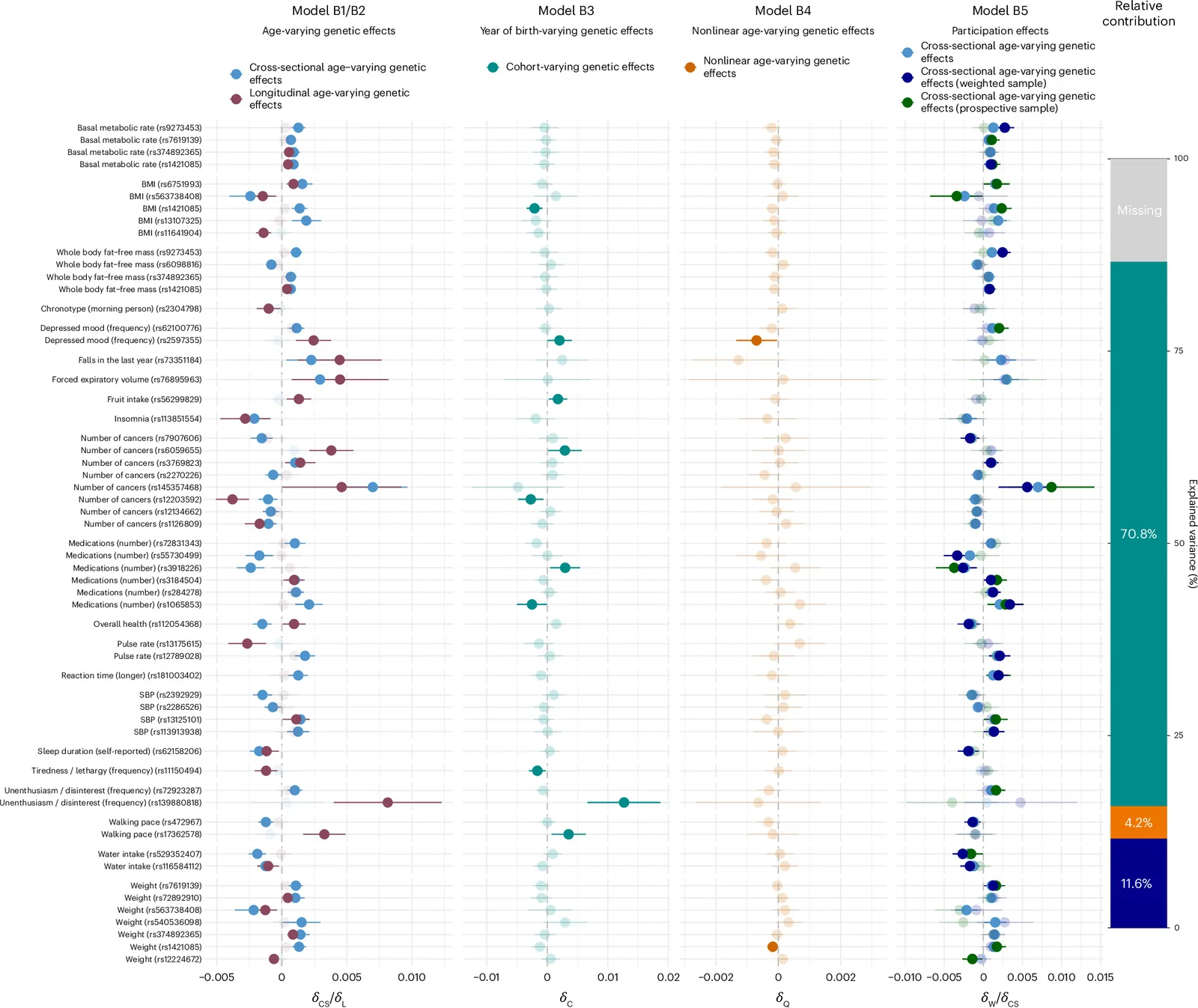
Factors contributing to differences in estimated age-varying genetic effects. The plots for models B1/B2 show the age-varying genetic effects obtained from cross-sectional analyes (${\delta }_{\mathrm{CS}}$ in model B1, highlighted in light blue) and longitudinal analyses (${\delta }_{\mathrm{L}}$ in model B2, highlighted in red). The plot for model B3 shows the cohort-varying genetic effects obtained from model B3 (${\delta }_{\mathrm{C}}$, in cyan). The plot for model B4 shows the nonlinear (quadratic) age-varying genetic effects (model B4, ${{\rm{\delta }}}_{\mathrm{Q}}$, in orange). The plot for model B5 contrasts the baseline participation adjusted cross-sectional age-varying genetic effects (obtained from model B5, ${\delta }_{\mathrm{W}}$, in dark blue) to cross-sectional age-varying genetic effects obtained from standard regression analysis in the UKB baseline sample (in light blue) and the UKB longitudinal sample (in green). The error bars represent the 95% confidence intervals. Estimates shown in more saturated colors indicate nominally significant effects (P<0.05) based on two-sided tests. The bar plot to the right indicates how much of the variance (in %) in the discrepancies between longitudinal and cross-sectional estimates (${\delta }_{\mathrm{L}}-{\delta }_{\mathrm{CS}}$) was accounted for by cohort-varying genetic effects (in cyan), nonlinear age-varying genetic effects (in orange) and selective participation (in dark blue). The source data used in this figure can be found in Supplementary Data 3.

The direction of age-varying effects was largely consistent between cross-sectional and longitudinal designs, but the magnitude of effects showed less agreement. Although a significant linear relationship was observed between cross-sectional (${\delta }_{\mathrm{CS}}$) and longitudinal (${\delta }_{\mathrm{L}}$) estimates (${\alpha }_{1}=$ 0.61, P= 4.17 × 10^−5^; obtained from ${\delta }_{\mathrm{L}}\sim {\alpha }_{0}+{\alpha }_{1} {\delta }_{\mathrm{CS}}$), the slope was significantly different from one (*t* = −2.85, P= 0.0062), indicating that cross-sectional estimates were systematically larger in magnitude than longitudinal estimates (Supplementary Fig. 3). To better understand these discrepancies, we next examined factors contributing to differences in estimated age-varying genetic effects, focusing on cohort-varying genetic effects (Fig. 5, model B3, in up to N= 69,178), nonlinear age-varying genetic effects (Fig. 5, model B4, up to N= 13,851) and selective participation (Fig. 5, model B5, in up to ${N}_{\mathrm{eff}}=$ 102,148). The source data used in Fig. 5 are provided in Supplementary Data 3.

Suggestive gene-by-cohort effects (that is, genetic effects that vary across the year of birth) were observed for 10 of the 57 examined variants (${\delta }_{\mathrm{C}}\ne 0$ at P<0.05). Together, these year-of-birth-dependent effects explained 70.8% of the variance in discrepancies between cross-sectional and longitudinal estimates, representing the principal contributor to the observed differences between designs. Notably, for several variants (for example, rs2597355 on depression, rs56299829 on fruit intake and rs17362578 on walking pace), confounding due to year of birth interactions resulted in a reversal of the estimated direction of age-varying genetic effects when using cross-sectional models. Of note, quantifying the contributions of these factors relies on important assumptions about the underlying causal pathways (for example, the absence of period-varying genetic effects). These assumptions are discussed further in the Discussion and in Supplementary Figs. 4 and 5.

We did find some evidence of nonlinear age-varying genetic effects for two of the 57 examined variants (${\delta }_{\mathrm{Q}}\ne 0$ at P<0.05). Although this phenomenon impacts both cross-sectional and longitudinal interaction estimates, it does not necessarily lead to discrepancies. In line with this argument, deviations from linearity represented only a negligible source of effect discrepancies between the two designs, accounting for 4.2% of the variance. Of note, because these estimates are derived from smaller subsets of the UKB and are, thus, subject to greater statistical larger estimation error, the variance component may be underestimated due to regression dilution^25^. To quantify this bias, we calculated the dilution ratio ($\lambda \in [0,1]$), where lower values denote greater attenuation. For the three factors considered (cohort, age^2^ and participation), nonlinear age-varying effects exhibited the largest dilution (λ= −0.43). After correction for attenuation bias (${R}_{\mathrm{corrected}}^{2}={R}_{\mathrm{observed}}^{2}/\lambda$), the variance explained by nonlinear age effects increased from 4.2% to −8.57% (Supplementary Fig. 6 and corresponding Supplementary Note).

More relevant in explaining effect discrepancies was bias due to selective participation (11.6% explained variance). Although selection bias can affect both designs, the nature of this bias may differ between approaches. Cross-sectional estimates are particularly affected by selective participation at baseline, which can impact findings if the initial sample is non-representative. By contrast, longitudinal models account for such time-invariant selection mechanisms (for example, baseline phenotype levels) but assume that continued participation is independent of time-varying factors (for example, changes in phenotype)^26^. Because different selection pressures may act during the initial recruitment and follow-up, participation bias can contribute to discrepancies between cross-sectional and longitudinal results.

When fitting the same set of models to polygenic effects, the findings mirrored those observed at the SNP level. Specifically, polygenic score (PGS)-by-gene effects were identified for anthropometric traits and metabolic indicators (BMI, weight and basal metabolic rate), with consistent directions of association but variation in effect magnitudes. When examining predictors of these discrepancies, cohort confounding emerged as the most relevant contributor, explaining 85.2% of the observed differences (Supplementary Fig. 7). Together, these results highlight that, for certain traits, PGS performance varies across contexts. This underscores the importance of taking into account both individual-level characteristics and social factors when evaluating the applicability and generalizability of PGS.

Finally, we illustrate the potential utility of age-varying genetic effects for causal inference within an MR framework, by examining whether longitudinal MR can recover the well-established adverse effect of BMI on systolic blood pressure (SBP). As shown in Supplementary Fig. 8, the estimated effects were directionally consistent with the known positive causal relationship between BMI and SBP when using age-varying genetic instruments. However, estimates from the longitudinal MR approach came with substantially greater uncertainty, due to the small number of available instruments and the weaker and noisier SNP−exposure associations.

## Discussion

Identifying age-varying genetic effects is essential for advancing understanding of the dynamic processes characterizing genetic influences on complex traits. Both cross-sectional and longitudinal approaches are designed to address this question but come with distinct strengths and limitations. In this work, we systematically evaluated the degree of convergence between estimates of age-dependent trait changes and age-dependent genetic effects derived from cross-sectional and longitudinal approaches, and we investigated factors that may contribute to any observed discrepancies between designs.

At the phenotypic level, cross-sectional age effects deviated from longitudinal estimates, with around 71% of the variance shared between the two approaches. The largest discrepancies were observed for behavioral and lifestyle traits such as smoking and alcohol use, mainly due to confounding by birth year (that is, cohort) effects, which can substantially alter or even reverse cross-sectional age estimates.

At the genotypic level, we observed high levels of consistency between cross-sectional and longitudinal approaches with respect to the direction of age-varying genetic effects. In both designs, attenuation, characterized by a decrease in genetic effects with advancing age, was a common pattern of interaction particularly for metabolic and well-being traits. This observation aligns with previous studies reporting decreasing heritability with advancing age during adulthood, as documented for BMI^27^ or depression^28^. Such a pattern suggests that accumulation of environmental exposures and age-related lifestyle changes (for example, medication use, changes in diet, sleep and occupational status) likely gain importance for those traits, thereby diminishing the relative contribution of genetics.

Under equivalence, cross-sectional and longitudinal analyses are expected to produce identical estimates of age-varying genetic effects. However, we observed only moderate agreement in the magnitude of these effects, suggesting that between-individual age differences may not accurately reflect within-individual age-related change. Among the factors examined, gene-by-cohort effects stood out as the main contributor, explaining the largest proportion of variance in effect discrepancies. Such effects pose a particular challenge for cross-sectional designs, which are unable to isolate the effects of biological aging from historical or societal changes that differentially impact birth cohorts. Of note, although we primarily examined cohort effects as a contributor to differences in the estimation of age-dependent genetic effects, it is important to recognize that such effects also represent meaningful and interpretable variation, capturing how historical or societal changes influence genetic effects across generations. This underscores the value of future research explicitly modeling gene-by-cohort interactions, which may provide important insights into contextual factors that shape genetic risk.

Selective participation was identified as the second most relevant contributor to differences in age-varying genetic effects obtained from the two designs. Although longitudinal designs are often regarded as more robust due to their ability to adjust for participation mechanisms unrelated to change^26^ (for example, initial selection into the sample), they are susceptible to selective dropout and survival effects^18,29^. By contrast, UKB cross-sectional analyses are affected by the well-documented healthy volunteer bias present at baseline^30–33^. Because the mechanisms driving participation at recruitment may differ from those affecting continued study involvement, these distinct selection pressures can contribute to discrepancies between cross-sectional and longitudinal results. It is important to note, however, that the bias observed here is most relevant to samples requiring active baseline and follow-up participation and may not generalize to prospective studies relying on passive data collection, such as those using routinely collected electronic health records.

Finally, we found that deviations from linearity contributed minimally to the differences in effect estimates between the two designs. Nevertheless, testing for nonlinear age effects remains important for two reasons. First, if nonlinear trajectories are present but not considered, both cross-sectional and longitudinal models assuming linearity will produce inaccurate estimates. Exploration of possible nonlinear patterns can, therefore, improve model specification and interpretability of the results. Second, when age-related change is nonlinear, the estimated linear slope becomes dependent on the underlying age distribution of the sample (Supplementary Fig. 1). Because the age ranges differ between the cross-sectional and longitudinal UKB samples (for example, a maximum baseline age of 69 years versus 86 years at follow-up), the observed age slopes are not directly comparable across designs, potentially leading to discrepant findings.

Overall, our findings highlight that longitudinal and cross-sectional designs are characterized by distinct strengths and limitations. Longitudinal cohort-heterogeneous designs allow for direct modeling of within-individual change over time, the identification of gene-by-cohort effects and the detection of nonlinear trajectories—insights that cannot be generated by cross-sectional designs but that are crucial for drawing valid conclusions about age-dependent effects. Conversely, cross-sectional analyses provided greater statistical power in our analyses of the UKB data, owing to their larger sample sizes and wider age ranges, thereby facilitating discovery of potentially age-varying genetic effects. Notably, this power advantage is specific to the current dataset, as longitudinal data characterized by minimal attrition and frequent follow-up assessments may offer similar or even greater statistical power while preserving the ability to model change over time. With the growing availability of whole-genome sequencing and longitudinal data (for example, electronic health records), future studies will also be able to extend these analyses to low-frequency and rare variants, potentially uncovering additional age-varying genetic effects.

Ultimately, neither design is without limitations, and the optimal choice will depend on the specific research question, data availability and tolerance for different sources of bias. Ideally, researchers may integrate both longitudinal and cross-sectional approaches, which offers a computationally feasible and more robust framework for identifying, quantifying and mitigating potential threats to the validity of dynamic genetic effects across the lifespan.

### Methodological considerations and analytical constraints

A central assumption of our analytical framework is that period effects—changes attributable to the specific calendar year in which individuals are assessed—are negligible. Such effects can bias age-related estimates if societal changes (for example, economic crises and public health interventions) affect all age groups simultaneously and would lead to misattribution of cohort effects in our longitudinal models. Because age, cohort and period are exactly collinear (age = period − cohort—that is, the ‘identification problem’^34^), it is statistically impossible to estimate all three effects simultaneously without constraints, typically by setting period effects to zero^35,36^. Overall, we think that the cohort effects identified in this study represent genuine cohort-specific influences rather than misattributed period effects. Theoretically, cohort effects capture exposures and experiences during formative developmental periods, which are likely to exert more lasting impacts on genetic associations than contemporaneous influences later in life. Supporting this, most gene-by-cohort effects detected here were observed for obesogenic traits and health behaviors, plausibly reflecting generational influences stemming from the 20th-century obesity epidemic, alongside improved public health initiatives and medical care^37–39^. Empirically, we conducted sensitivity analyses excluding participants whose follow-up occurred during or after the COVID-19 pandemic—the most profound recent environmental shock. Results showed high concordance with the full sample for both phenotypic (r= 0.98) and genotypic (r= 0.98) associations (Supplementary Fig. 4), suggesting that even substantial period disruptions had minimal impact on our findings.

As discussed in the Supplementary Information, these include scenarios in which genetic effects on a trait are mediated by an intermediate event as well as scenarios in which genetic effects on the event and on the trait operate independently. Distinguishing between these mechanisms has potentially important implications for model specification and interpretation and may require joint modeling of longitudinal traits and time-to-event outcomes in future work.

It is also important to note that the identified age-varying genetic effects may operate through indirect pathways, and a deeper understanding of these mechanisms will require more tailored modeling approaches. For example, longitudinal trajectories can be substantially altered by major health events occurring during follow-up, such as disease onset. When such events are themselves genetically influenced, explicitly modeling them can facilitate more precise mechanistic interpretations. As detailed in Supplementary Fig. 8, this includes (1) mediating pathways, in which genetic effects on a trait operate through an intermediate event, and (2) independent pathways, in which genetic effects on the event and the trait act separately.

Finally, we explore the implications of age-varying genetic effects for causal inference using MR. Although presented for illustration only, these analyses demonstrate how longitudinally derived genetic effects could, in principle, be incorporated into MR frameworks to test if changes in an exposure translate into changes in an outcome. In practice, adequately powered longitudinal MR will require substantially larger genome-wide association studies to identify a sufficient number of strong instruments indexing change. Moreover, longitudinal MR estimates are interpretable only under additional assumptions, namely that time-varying genetic effects are independent of lifetime genetic effects and that selected instruments do not exhibit higher-order age-dependent effects (for example, gene-by-age^2^ interactions). Evaluating these (testable) assumptions should, therefore, be an integral part of sensitivity analyses when applying this approach.

## Methods

We included 498,845 UKB individuals aged 40−69 years who were first seen between 2006 and 2010 in one of the 22 assessment centers, corresponding to a cohort of individuals born between 1937 and 1970. The UKB was approved by the National Health Service North-West Center Research Ethics Committee (REC no. 16/NW/0274), and this research was conducted under application number 16389. From the baseline sample, we selected up to 99,459 individuals who were followed-up in repeat assessment(s) carried out between 2012 and 2024 (mean follow-up duration, 11 years; range, 2−18 years). The UKB was approved by the National Health Service North-West Center Research Ethics Committee (REC no. 16/NW/0274), and this research was conducted under application number 16389.

We selected 31 non-binary phenotypes with at least 50,000 non-missing repeat measurements per phenotype, tapping into dimensions of cognition (for example, reaction time), health (for example, SBP and forced expiratory volume), anthropometrics (for example, BMI and whole body fat−free mass) or lifestyles (for example, smoking and alcohol consumption). Excluded were all phenotypes with only one assessment available, as parts of our modeling approach relied on the use of a subset of UKB participants with at least three measurements to explore possible nonlinear age effects (see below). As a result, we included only phenotypes that were reassessed as part of the UKB neuroimaging follow-up assessments (for example, excluding biomarker measures such as low-density lipoprotein (LDL))^40^. Each phenotype ${P}_{{\mathrm{t}}}$ assessed at a given timepoint t was standardized prior to the inclusion in the analytical models ($\frac{{P}_{t}-{\mu }_{0}}{{\sigma }_{0}}$), based on their baseline mean (${\mu }_{0}$) and standard deviation (${\sigma }_{0}$).

### Statistics and reproducibility

No statistical methods were used to predetermine sample sizes, but our sample sizes are similar to those reported in previous publications^18,19^. The analyses are reproducible from the publicly available analytical scripts.

#### Cross-sectional and longitudinal age effects

We first performed a series of analyses to assess factors influencing the degree of convergence between age effects obtained from cross-sectional (between-subject) comparisons (${\beta }_{\mathrm{CS}}$ in model A1; see below) and longitudinal (within-subject) comparisons (${\beta }_{\mathrm{L}}$ in A2), focusing on cohort effects (${\beta }_{\mathrm{C}}$ in model A3), nonlinear (quadratic) age effects (${\beta }_{\mathrm{Q}}$ in model A4) and selective participation (weighted ${\beta }_{\mathrm{W}}$ in model A5) (Fig. 2):

1 $${P}_{0j}={\alpha }_{0}+{\beta }_{\mathrm{CS}} {\mathrm{age}}_{0j}+{\varepsilon }_{j}$$

2 $${P}_{{tj}}={\alpha }_{0}+{\beta }_{\mathrm{L}} {\mathrm{age}}_{c,{tj}}+u_{j}+{\varepsilon }_{{tj}}$$

3 $${P}_{{tj}}={\alpha }_{0}+{\beta }_{\mathrm{L}} {\mathrm{age}}_{{tj}}+{\beta }_{\mathrm{C}} {\mathrm{cohort}}_{j}+u_{j}+{\varepsilon }_{{tj}}$$

4 $${P}_{{tj}}={\alpha }_{0}+{\beta }_{\mathrm{L}} {\mathrm{age}}_{c,{tj}}+{\beta }_{\mathrm{Q}} {\mathrm{age}}_{c,{tj}}^{2}+u_{j}+{\varepsilon }_{{tj}}$$

5 $${P}_{0j}={\alpha }_{0}+{\beta }_{\mathrm{W}} {\mathrm{age}}_{0j}+{\varepsilon }_{j}$$

${P}_{{tj}}$ represents a given phenotype at timepoint t (with t=0 representing the phenotype assessed at baseline) for individual j. Cross-sectional age effects were obtained from a standard linear regression model (A1), regressing the baseline phenotype (${P}_{0j}$) on baseline age (${\mathrm{age}}_{0j}$), where ${\alpha }_{0}$ denotes the intercept. This model was applied to all UKB participants who had non-missing phenotype values recorded at their initial assessment center visit, conducted between 2006 and 2010 (up to N= 498,845). Models using repeat measurements (A2–A4) were tested by including age as a fixed effect in linear mixed-effects models implemented in lme4 (ref. ^41^), where $u_{j}$ denotes the random intercept to account for within-individual clustering and ${\varepsilon }_{{tj}}$ denotes the residual error. In model 2, we included only individuals with at least one follow-up assessment (up to N= 99,459), and model 4 was restricted to individuals with at least three assessment waves (up to N= 19,374), allowing for the estimation of quadratic age effects. In those two models, ${\mathrm{age}}_{c,{tj}}$ represents the person-mean centered age at time t for individual j (${\mathrm{age}}_{c,{tj}}={\mathrm{age}}_{{tj}}-\overline{{\mathrm{age}}_{j}}$), which distinguishes within-individual age effects from between-individual variation^42–45^. Specifically, age centering eliminates bias in the age estimate that results from systematic variation in mean age across birth years, thereby removing confounding due to cohort effects^43,46,47^. Cohort effects (model A3, applied in up to N= 99,459 individuals with at least one follow-up assessment) reflect between-individual differences in birth year and were modeled by including both (uncentered) age and year of birth of individual j as fixed effects. Age was not centered in this model, as person-mean centering removes between-person variance, thereby precluding the estimation of birth year effects^43,46^. To assess nonlinear age effects over time (model A4), analyses were restricted to individuals with at least three assessment waves, allowing for the estimation of quadratic age effects. Finally, the impact of selective participation was evaluated by comparing age effects across samples with varying levels of representativeness. Inverse probability weighting (model A5) implemented in the ‘survey’^48^ package was applied to obtain weighted age coefficients (${\beta }_{\mathrm{W}}$) adjusted for selective baseline participation, using previously derived sampling weights^31^. These weighted effects were then compared to those from the unweighted baseline and prospective sample, to assess whether age effects vary as a function of sample representativeness.

To assess the relative contribution of cohort, nonlinear and participation effects to discrepancies between longitudinal (${\beta }_{\mathrm{L}}$) and cross-sectional (${\beta }_{\mathrm{CS}}$) age estimates, we modeled the effect size differences as a function of these factors (${\beta }_{\mathrm{L}}-{\beta }_{\mathrm{CS}}\sim {\alpha }_{0}+{\alpha }_{1} {\beta }_{\mathrm{C}}+{\alpha }_{2} {\beta }_{\mathrm{Q}}+{\alpha }_{3} {\beta }_{\mathrm{P}}$). Here, ${\beta }_{\mathrm{P}}$ indexes participation effects, defined as the difference in cross-sectional age estimates obtained from a representative (weighted estimate, ${\beta }_{\mathrm{W}}$ in model A5) versus a less representative (unweighted estimate, ${\beta }_{\mathrm{C}}$ in model A1, obtained in the prospective sample) (${\beta }_{\mathrm{P}}={\beta }_{\mathrm{W}}-{\beta }_{\mathrm{C}}$). The proportion of variance explained by each predictor (${R}^{2}$) was calculated using the ‘lmg’ method in the relaimpo^49^ package, following the approach developed by Lindeman et al.^50^.

Because the estimates for each factor (${\beta }_{\mathrm{C}}$, ${\beta }_{\mathrm{Q}}$, ${\beta }_{\mathrm{P}}$) were derived from different subsets of UKB data with varying sample sizes, some are subject to larger estimation error, potentially leading to an underestimation of variance components due to regression dilution^25^. To account for this bias, we calculated the dilution ratio ($\lambda \in [0,1]$) for each factor, where lower λ values indicate greater attenuation. Corrected variance contributions (${R}_{\mathrm{corrected}}^{2}$) were obtained by dividing the observed ${R}^{2}$ values by λ (${R}_{\mathrm{corrected}}^{2}={R}_{\mathrm{observed}}^{2}/\lambda$). The dilution ratio was computed as $\lambda =\frac{{V}_{\mathrm{total}}-{V}_{\mathrm{error}}}{{V}_{\mathrm{total}}}$, with ${V}_{\mathrm{total}}=\frac{1}{n-1}\mathop{\sum }\nolimits_{i=1}^{n}{({\hat{\theta }}_{i}-\mu )}^{2}$ and ${V}_{\mathrm{error}}=\frac{1}{n}\mathop{\sum }\nolimits_{i=1}^{n}SE{({\hat{\theta }}_{i})}^{2}$. $\hat{\theta }$ denotes the vector of estimated coefficients and $\mu =\frac{1}{n}\mathop{\sum }\nolimits_{i=1}^{n}{\hat{\theta }}_{i}$ (see Supplementary Note for details).

### Identification of age-varying genetic effects

To identify age-varying genetic effects, we implemented a two-step genome-wide strategy that integrates marginal, cross-sectional and longitudinal models to detect age-varying genetic effects^19^, as illustrated in Fig. 3a. In the first step, we conducted genome-wide scans for marginal genetic effects (${\gamma }_{M}$ in model 6), cross-sectional gene-by-age interaction effects (${\delta }_{\mathrm{CS}}$ in model 7) and genetic effects on change (${\delta }_{\mathrm{L}}$ in model 8).

6 $${P}_{0j}={\textbf{W}} {\boldsymbol{\alpha }}+{\beta }_{\mathrm{CS}} {\mathrm{age}}_{0j}+{\gamma }_{\mathrm{M}} G_{j}+{\varepsilon }_{j}$$

7 $${P}_{0j}={\textbf{W}} {\boldsymbol{\alpha }}+{\beta }_{\mathrm{CS}} {\mathrm{age}}_{0j}+{\gamma }_{\mathrm{M}} G_{j}+{\delta }_{\mathrm{CS}} ({\mathrm{age}}_{0j}\times G_{j})+{\varepsilon }_{j}$$

8 $${P}_{{{\Delta }}_{j}}={\textbf{W}} {\boldsymbol{\alpha }}+{\delta }_{\mathrm{L}} G_{j}+{\varepsilon }_{j}$$

Here, $\textbf{W}$ denotes the matrix of covariates (including intercept, the first 20 genetic principal components, genotyping array and sex), and ${\boldsymbol{\alpha }}$ represents their corresponding coefficients. The outcome for model 8, ${P}_{{\Delta }_{j}}$, reflecting an individual’s annual rate of change in a given phenotype, is calculated as the difference between the most recent follow-up and baseline phenotype (${P}_{1}-{P}_{0}$), divided by follow-up duration (FU): ${P}_{{{\Delta }}_{j}}=\frac{{P}_{1}-{P}_{0}}{\mathrm{FU}}$. Further considerations regarding the assumptions and limitations of such ratio traits are provided in the Supplementary Note. To increase robustness, participants with fewer than 3 years of follow-up were excluded from the longitudinal model.

Genome-wide association analyses were conducted using REGENIE (version 3.2.6)^51^. First, a whole-genome regression model was fitted using ridge regression to account for local linkage disequilibrium patterns and relatedness among individuals. Quality-controlled SNPs were included in this step, with filtering criteria of minor allele frequency (MAF) ≥ 1%, Hardy−Weinberg equilibrium P $\ge 1\times {10}^{{-}15}$, genotyping rate ≥ 99%, exclusion of SNPs involved in inter-chromosomal linkage disequilibrium and additional linkage disequilibrium pruning (${R}^{2}$ threshold of 0.9, window size of 1,000 markers, step size of 100 markers)^51^. Second, association testing was performed using linear regression models that condition on predictions generated during the first step. This phase utilized the imputed UKB genotype data (version 3, imputed with the Haplotype Reference Consortium panel), considering variants with MAF > 1%. Analyses were restricted to participants of European genetic ancestry, after exclusion of individuals displaying high levels of missingness or extreme autosomal heterozygosity (as defined by UKB^52^). In REGENIE, we used the parameter ‘–interaction age –no-condtl’ to obtain the gene-by-age interaction (${\delta }_{\mathrm{CS}}$ in model 7) in addition to the (unconditioned) marginal genetic effects (${\gamma }_{\mathrm{M}}$ in model 6) within the same iteration. Genome-wide tests on age-related annual change (${\delta }_{\mathrm{L}}$ in model 8) were obtained from the standard REGENIE analysis without the specification of the interaction parameter.

In the final step, we identified candidate genetic variants by selecting all linkage-disequilibrium-independent SNPs that reached genome-wide significance ($P < 5\times {10}^{-8}$) in any of the three models of marginal effects (${\gamma }_{\mathrm{M}}$ in model 6), cross-sectional interaction effects (${\delta }_{\mathrm{CS}}$ in model 7) and/or effects on change (${\delta }_{\mathrm{L}}$ in model 8), after stringent clumping in plink^53^ (–clump-kb 10000, –clump-r2 0.001)^54^. From this set, we selected all Bonferroni-corrected significant variants (discovery threshold: $P < 0.05/{k}_{\mathrm{eff}}$) identified in the two models testing for age-varying genetic effects (${\delta }_{\mathrm{CS}}$ and ${\delta }_{\mathrm{L}}$, models 7 and 8), where ${k}_{\mathrm{eff}}$ denotes the number of genome-wide variants per trait identified in step 1 (Fig. 3a). Of note, although the Bonferroni correction using ${k}_{\mathrm{eff}}$ is based on dependent tests (as ${k}_{\mathrm{eff}}$ is derived from candidate variants identified across all three genome-wide models), its application to the age-varying models (cross-sectional and longitudinal) imposes a stricter discovery threshold, thus reflecting a conservative approach. The resulting list of genetic variants exhibiting age-varying effects was then used in downstream analyses evaluating the robustness of these findings.

#### Cross-sectional and longitudinal age-varying genetic effects

For the set of identified variants, we first evaluated whether the age-varying genetic effects obtained from cross-sectional and longitudinal analyses were consistent with respect to the direction and interpretation of the effects. To do this, we classified the interaction patterns as attenuation, intensification or crossover, based on the relationship between the marginal genetic effect and the age-varying effect. Specifically, attenuation was inferred if marginal and interaction effects had opposite signs (for example, a positive interaction effect with a negative main effect), whereas intensification was indicated by concordant signs (for example, both main and interaction effects negative). Effects were labeled as ‘crossover’ when a significant interaction effect was present in the absence of a significant main effect.

To investigate potential sources of discrepancy between cross-sectional and longitudinal estimates of age-varying genetic effects, we extended the previously described phenotypic models (A1–A5) by incorporating the genetic term ($G_{j}$) and estimated the relevant parameters for all candidate genetic variants with indicated age-varying effects. Specifically, we compared the age-varying genetic effects obtained from the cross-sectional (${\delta}_{\mathrm{CS}}$, model B1) and longitudinal (${\delta}_{\mathrm{L}}$, model B2) approach and fitted a series of additional models designed to assess the influence of variations in genetic effects at the level of cohort differences ($\delta_{\rm{C}}$, model B3), nonlinear (quadratic) age effects (${\delta}_{\mathrm{Q}}$, model B4) and selective participation ($\delta_{\rm{W}}$, model B5). The parameters of interest were estimated as follows:

B1 $$P_{0j} = \textbf{W} \boldsymbol{\alpha} + \beta_{\textrm{CS}} \mathrm{age}_{0j} + \gamma_{\textrm{M}} G_j +{\delta_{\mathrm{CS}}} (\mathrm{age}_{0j} \times G_j) + \varepsilon _{j}$$

B2 $$P_{tj} = \textbf{W} \boldsymbol{\alpha} + \beta _{\textrm{L}} \mathrm{age}_{c,tj} + \gamma_{\textrm{M}} G_j +{\delta_{\mathrm{L}}} (\mathrm{age}_{c,tj}\times G_j) + u_j + \varepsilon _{tj}$$

B3 $$\begin{array}{l}{P}_{{tj}}={\bf{{W}}} {\boldsymbol{\alpha }}+{\beta }_{\mathrm{L}} {\mathrm{age}}_{{tj}}+{\beta }_{\mathrm{C}} {\mathrm{cohort}}_{j}+{\beta }_{\mathrm{L}\times \mathrm{C}} ({\mathrm{age}}_{{tj}}\times {\mathrm{cohort}}_{j})\\\qquad+{\gamma }_{\mathrm{M}} G_{j}+{\delta }_{\mathrm{L}} ({\mathrm{age}}_{{tj}}\times G_{j})+{\delta }_{\mathrm{C}} ({\mathrm{cohort}}_{j}\times G_{j})+u_{j}+{\varepsilon }_{{tj}}\end{array}$$

B4 $$\begin{array}{l}P_{tj} = \textbf{W} \boldsymbol{\alpha} + \beta_1 \mathrm{age}_{c,tj} + \beta_2 \mathrm{age}_{c,tj}^2 + \gamma G_j + \delta_{\mathrm{L}} (\mathrm{age}_{c,tj} \times G_j)\\\qquad+{\delta_{\mathrm{Q}}} (\mathrm{age}_{c,tj}^2 \times G_j) + u_j + \varepsilon_{tj}\end{array}$$

B5 $${P}_{0j}={\bf{{W}}} {\boldsymbol{\alpha }}+{\beta }_{W} {\mathrm{age}}_{0j}+{\gamma }_{\mathrm{M}} G_{j}+{\delta }_{\mathrm{W}} ({\mathrm{age}}_{0j}\times G_{j})+{\varepsilon }_{j}$$

All model parameters were estimated in R in a sample of unrelated UKB participants, applying ordinary least squares regression (model B1), linear mixed-effects models (10–B4) and weighted least squares regression (model B5). Of note, the age-by-cohort interaction term in model B3 (${\beta }_{\mathrm{L}\times \mathrm{C}}$) was added to appropriately control for age when estimating cohort-varying genetic effects^55^. As in the phenotypic analyses, we quantified the relative contribution of each factor to the observed differences in age-varying genetic effects between the two designs by modeling $({\delta }_{\mathrm{L}}-{\delta }_{\mathrm{CS}})\sim {\alpha }_{0}+{\alpha }_{1} {\delta }_{\mathrm{C}}+{\alpha }_{2} {\delta }_{\mathrm{Q}}+{\alpha }_{3} {\delta }_{\mathrm{P}}$, where ${\delta }_{\mathrm{P}}$ indexes participation effects.

Furthermore, to evaluate the implications of cross-sectional versus longitudinal modeling for PGS applications, we reestimated models B1–B5 replacing single-variant predictors ($G_{j}$) with trait-specific PGSs. We then compared the resulting age-varying polygenic effects across the different models (see Supplementary Note for details).

Finally, we illustrate the implications of age-varying genetic effects for causal effect estimation using MR, a widely applied method that leverages genetic instruments to estimate the causal effect of an exposure on an outcome^56^. Although most MR studies rely on genome-wide association estimates derived from time-invariant models, there are several reasons why a longitudinal MR framework may be informative. First, standard MR estimates are typically interpreted as reflecting lifelong genetic predisposition to higher exposure levels^57^ rather than the effect of modifying the exposure over a defined period. A longitudinal framework, estimating the effect of annual change in the exposure on annual change in the outcome, may, therefore, offer an interpretation more closely aligned with intervention effects. Second, genetic influences on mean levels and on longitudinal change have been shown to be mostly distinct^18^. Consequently, potential violations of MR assumptions, such as horizontal pleiotropy or confounding, are likely to differ between time-invariant and time-varying genetic instruments. As a result, potential violations of MR assumptions (for example, horizontal pleiotropy or confounding) may differ between time-invariant and time-varying instruments. Triangulating across these approaches may, therefore, strengthen causal inference. Details on the longitudinal MR approach employed here can be found in the Supplementary Note.

### Reporting summary

Further information on research design is available in the Nature Portfolio Reporting Summary linked to this article.

## Supplementary information

Supplementary Information Supplementary Figs. 1−8, Note and references.

Reporting Summary

Peer Review File

Supplementary Table Supplementary Tables 1−4.

## Data Availability

All data underlying this study are publicly available to researchers via the UKB at https://www.ukbiobank.ac.uk/. The genome-wide summary statistics generated as part of this work are deposited in the NHGRI-EBI GWAS Catalogue at https://www.ebi.ac.uk/gwas/ under accession codes GCST90994039-GCST90994100.

## Code availability

The following software was used to run the analyses:

• REGENIE^51^ (https://github.com/rgcgithub/regenie)

• bigsnpr^58^ (https://privefl.github.io/bigsnpr/)

• gwasrapidd^59^ (https://rmagno.eu/gwasrapidd/)

• otargen^60^ (https://amirfeizi.github.io/otargen/)

• survey^48^ (CRAN)

• lme4 (ref. ^41^) (CRAN)

• PRSice-2 (ref. ^61^) (https://choishingwan.github.io/PRSice/)

All analytical scripts are available at https://github.com/TabeaSchoeler/TS2024_UKBBageEffect.
