# Supplementary material for: Design and model choices shape inference of age-varying genetic effects on complex traits
Source: Nat Aging. 2026 Oct 6;6(10):2253–64. doi: 10.1038/s43587-026-01232-w (PMC13661259; doi:10.1038/s43587-026-01232-w)
Supplement: Supplementary file 1 [file 43587_2026_1232_MOESM1_ESM.pdf]

nature aging

Supplementary information https://doi.org/10.1038/s43587-026-01232-w

Design and model choices shape inference of age-varying genetic effects on complex traits

In the format provided by the authors and unedited

Supplement File

Contents

Supplementary Figure 1. Illustration of non-linear age effects . . . .
Supplementary Figure 2. Correction for selective participation . . . .
Supplementary Figure 3. Consistency of age-varying genetic effects obtained from cross-sectional and longitudinal analyses . . . .
Supplementary Figure 4. Age-varying genetic effects obtained in individuals participating prior to the COVID-19 pandemic . . . .
Supplementary Figure 5. Structural causal models of pathways influencing health trajectories . . . .
Supplementary Figure 6. Quantifycation of regression dilution bias . . . .
Supplementary Figure 7. Implications for polygenic score approaches . . . .
Supplementary Figure 8. Implications for Mendelian Randomization analyses . . . .
Supplementary note on change ratios . . . .
References . . . .

Supplementary Figure 1. Illustration of non-linear age effects

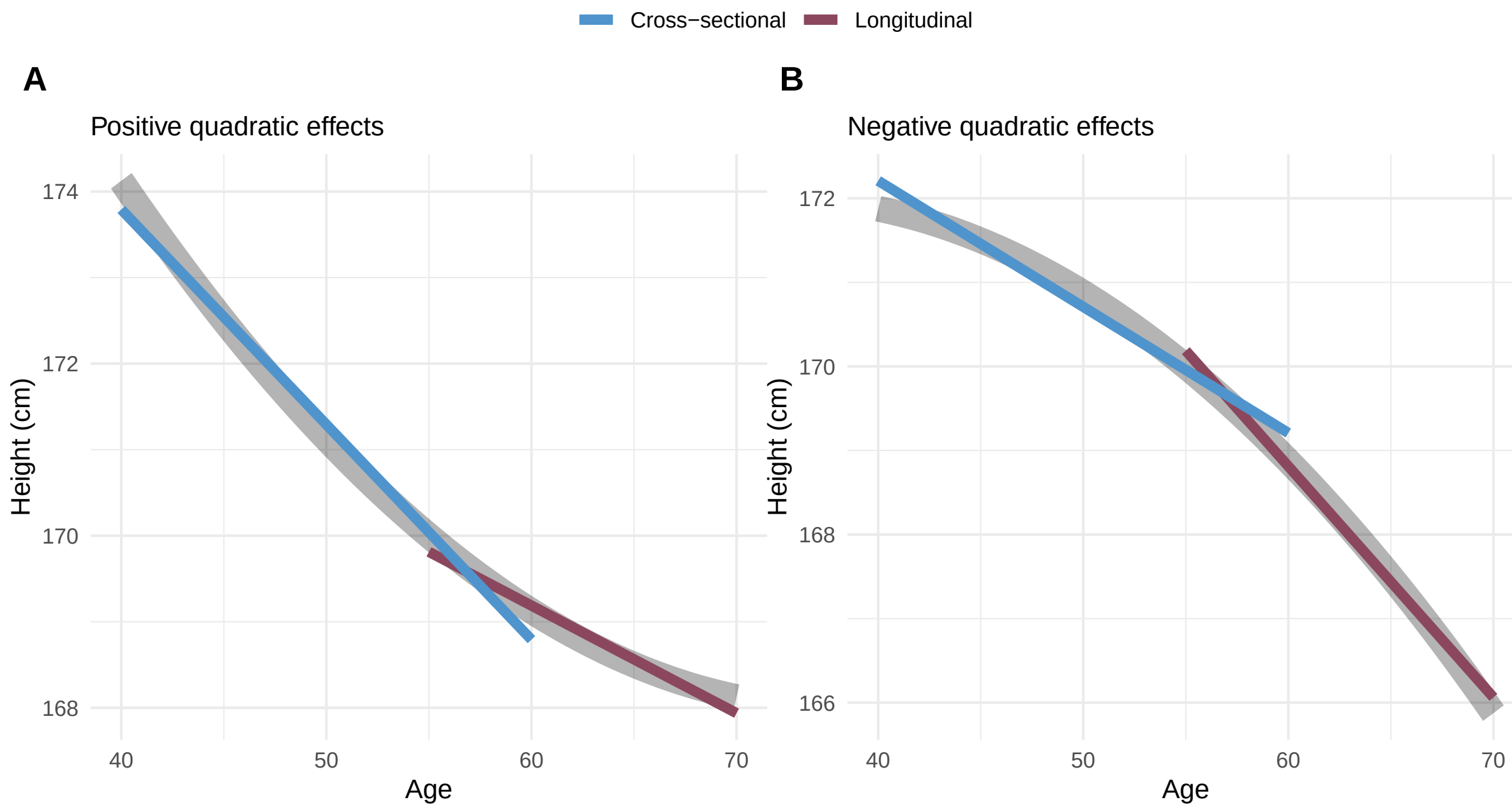

Supplementary Figure 1 illustrates the impact of non-linear (quadratic) age effects on cross-sectional and longitudinal slope estimates. The figure depicts simulated age trajectories for an outcome variable (e.g., height) as a function of age, including both linear and quadratic (nonlinear) age effects. The underlying outcome is modeled as height $= 170 + \beta_1 \cdot \text{age}_c + \beta_1 \cdot \text{age}_c^2$, where β_1 is a linear age effect, β_2 is a quadratic age effect (set to either positive or negative for illustrative purposes) and age_c denotes age centered at the mean. Two age ranges were selected to represent different cross-sectional (ages 40–60) and longitudinal (ages 55–70) study windows. Linear regression slopes are fit separately to each age window, representing the estimated age effect from cross-sectional (in blue) and longitudinal (in red) approaches, respectively. The plot highlights how the estimated slope (i.e., age effect) depends on the underlying age distribution when non-linear effects are present, leading to potentially discrepant estimates between study designs.

Supplementary Figure 2. Correction for selective participation

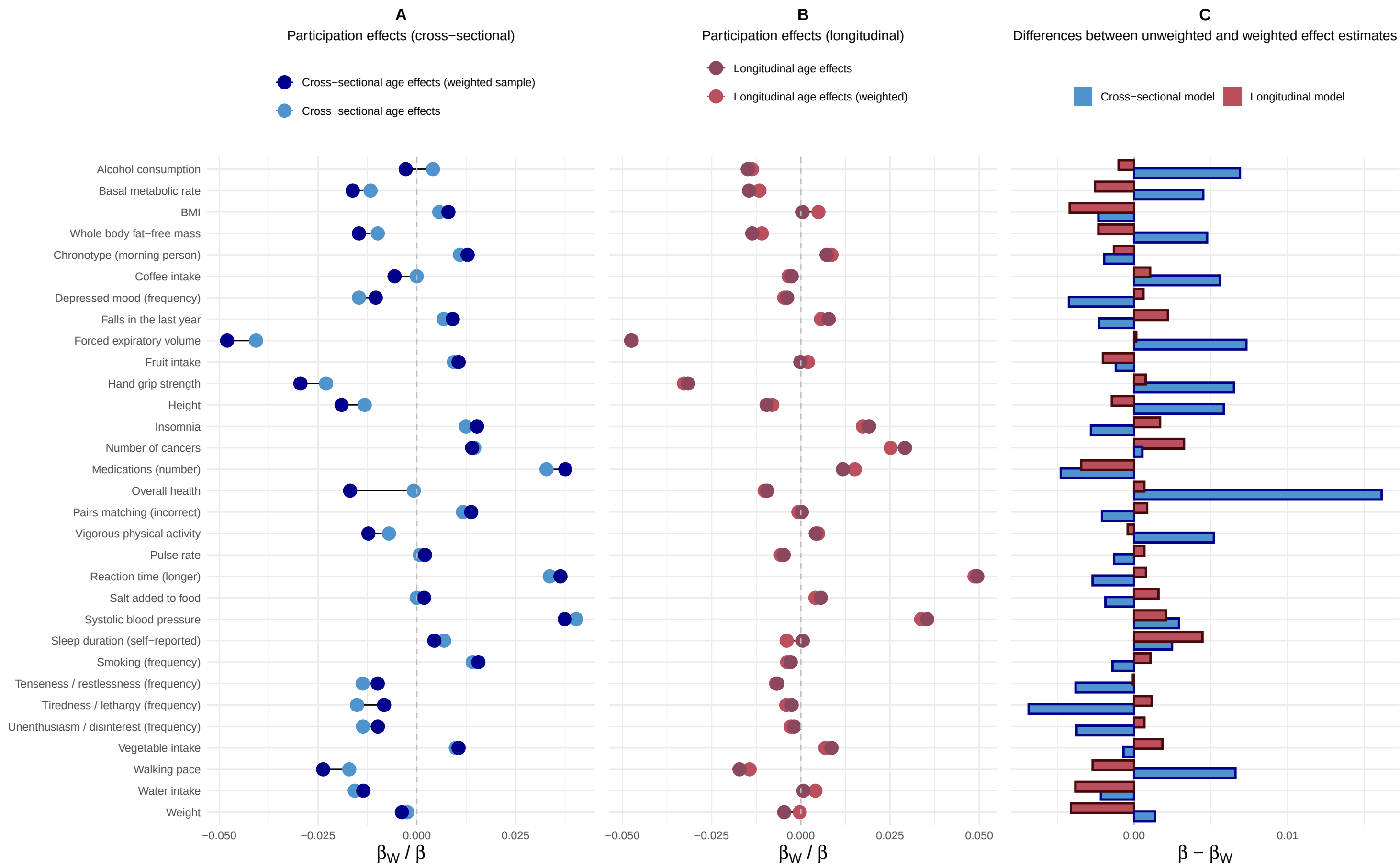

To assess the impact of selective participation, including selective baseline participation and selective follow-up participation, we applied inverse probability weighting to account for non-representativeness arising from these mechanisms.

For baseline participation, we used previously generated UK Biobank participation probabilities designed to correct for selective baseline participation[1]. These sampling weights were derived using a LASSO regression model predicting participation in baseline assessments (1 = participated; 0 = representative reference sample), based on the same 14 harmonized predictors described previously[1]. For follow-up participation, we generated a second set of weights using the same approach. Specifically, we estimated the probability of participation in prospective follow-up assessments (1 = participated in follow-up; 0 = representative reference sample) using a LASSO regression model with the identical set of predictors.

For baseline participation, we used previously generated UK Biobank participation probabilities designed to adjust for selective baseline participation[1]. The sampling weights were generated by fitting a LASSO regres-

sion models predicting UK Biobank participation (1 = participated in baseline assessments; 0 = representative reference sample), based on the same 14 harmonized predictors described previously[1]. For follow-up participation, we generated a second set of weights using the same appraoch, deriving the probability weights from a model a predicting follow-up participation (1 = participated in prospective follow-up; 0 = representative reference sample),

For the cross-sectional models, baseline sampling weights were incorporated using weighted least squares regression implemented in the R package *survey*[2]. For the longitudinal models applied to the prospective UK Biobank sample, follow-up sampling weights were incorporated into weighted linear mixed-effects models using the R package *svylme*[3].

Supplementary Figure 2 summarizes weighted and unweighted age effects from cross-sectional analyses (Panel **A**) and longitudinal analyses (Panel **B**). Panel **C** displays the absolute difference between the weighted and unweighted estimates, shown separately for the cross-sectional (in blue) and longitudinal models (in red). Overall, weighting had a smaller impact on longitudinal than on cross-sectional estimates ($\text{mean}_{|\beta-\beta_w|}$=0.0018 versus $\text{mean}_{|\beta-\beta_w|}$=0.004, respectively). However, for several traits, the impact of weighting was larger in the longitudinal models than in the cross-sectional models (e.g., BMI, number of cancers), possibly indicating that these traits are more relevant for dropout during follow-up than of initial baseline participation.

Supplementary Figure 3. Consistency of age-varying genetic effects obtained from cross-sectional and longitudinal analyses

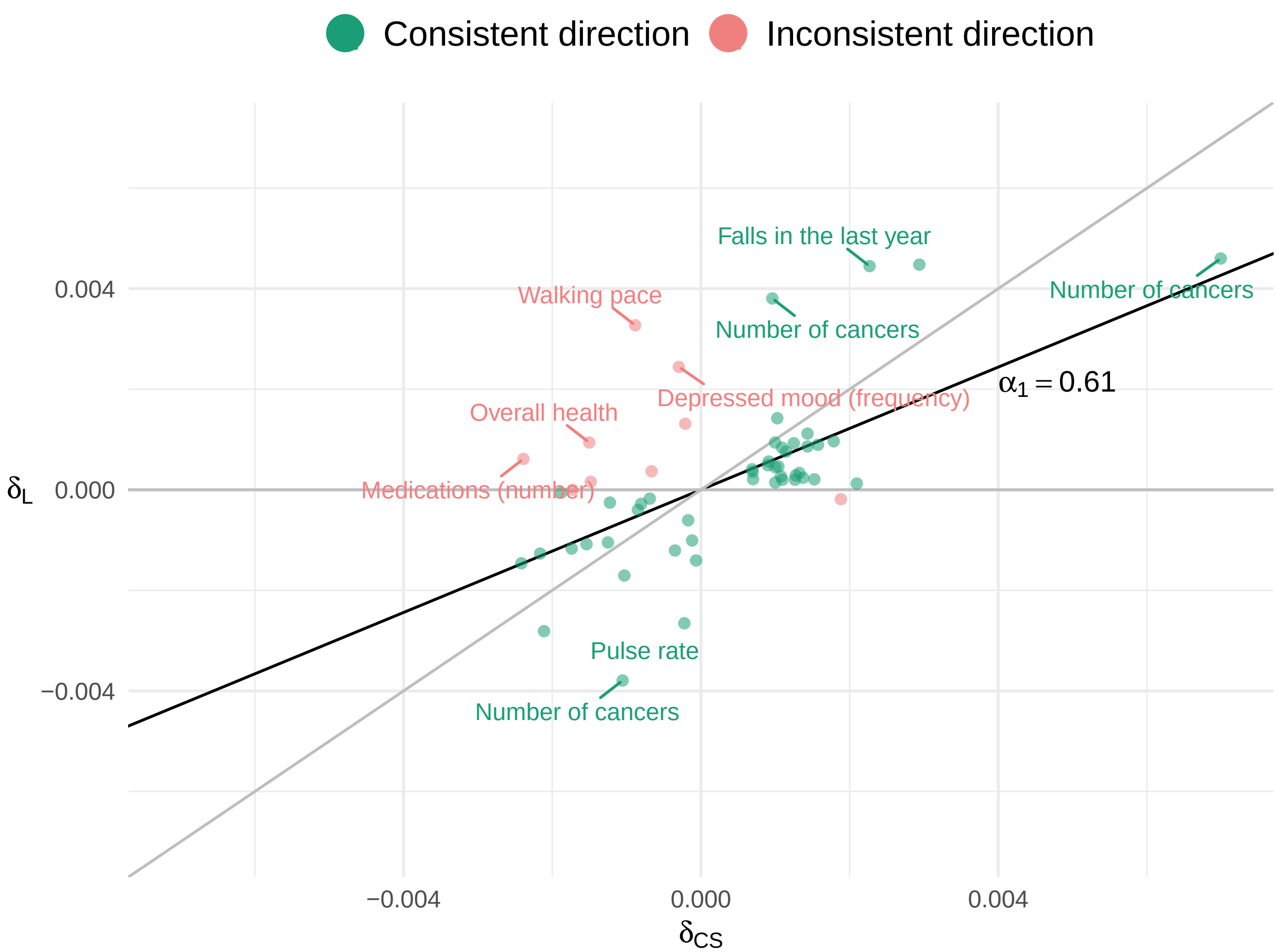

Supplementary Figure 3 plots the effect sizes of all identified variants with age-varying effects obtained from cross-sectional analyses (δ_{CS}, on the x-axis) and longitudinal models (δ_{L}, on the y-axis). The regression coefficient (α_1) was obtained from the model $\delta_{\mathrm{L}} \sim \alpha_0 + \alpha_1 \cdot \delta_{\mathrm{CS}}$, were α_0 denotes the intercept. The results show that there was a significant linear relationship between the cross-sectional and longitudinal age-varying effects ($\alpha_1 = 0.61$, $P = 4.17\text{e-}05$). However, the slope was significantly different from one (t = -2.85, $P = 0.006$), indicating that cross-sectional estimates were systematically inflated compared to the longitudinal estimates.

Supplementary Figure 4. Age-varying genetic effects obtained in individuals participating prior to the COVID-19 pandemic

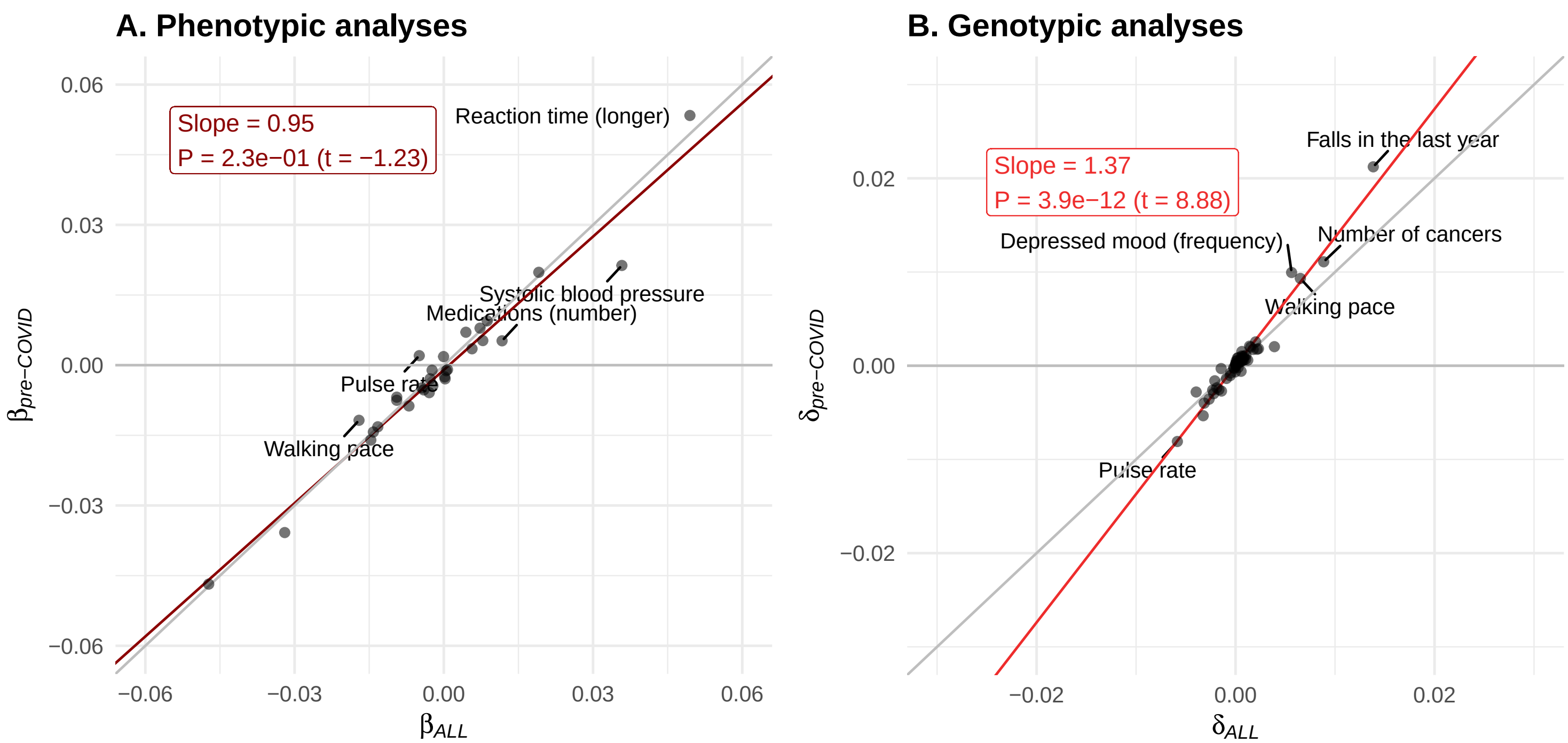

Supplementary Figure 4A plots the longitudinal age effects (β) estimated for all 31 included traits (obtained from $P_{tj} = \alpha_0 + \beta \cdot \text{age}_{c,tj} + u_j + \varepsilon_{tj}$, see model A1 in the main manuscript). Estimates are shown for the full UK Biobank sample (β_{ALL}) and for a restricted subsample that excludes participants whose follow-up occurred during or after the COVID-19 pandemic (after 31st December 2019; $\beta_{\text{pre-COVID}}$). The text box in the plot shows the slope (α_1) from the regression $\beta_{\text{ALL}} \sim \alpha_0 + \alpha_1 \cdot \beta_{\text{pre-COVID}}$, where α_0 is the intercept. The associated P-value was obtained from a two-sided t-test, assessing whether the slope significantly differed from one.

Supplementary Figure 4B displays the effect sizes for all variants showing age-varying effects, estimated from longitudinal genome-wide models ($P_{\Delta_j} = \mathbf{W} \cdot \alpha + \delta \cdot G_j + \varepsilon_j$, see main manuscript) using REGENIE. As above, estimates are shown for both the full sample (δ_{ALL}) and the pre-pandemic subsample ($\delta_{\text{pre-COVID}}$). The text box includes the slope (α_1) from the regression $\delta_{\text{ALL}} \sim \alpha_0 + \alpha_1 \cdot \delta_{\text{pre-COVID}}$, and the P-value testing if the slope significantly differed from one.

Overall, effect estimates derived from participants assessed before the COVID-19 pandemic were highly concordant with those from the full sample, for both phenotypic ($r = 0.98$, $P = 4.8\text{e-}20$) and genotypic ($r = 0.98$, $P = 2.9\text{e-}37$) associations.

Supplementary Figure 5. Structural causal models of pathways influencing health trajectories

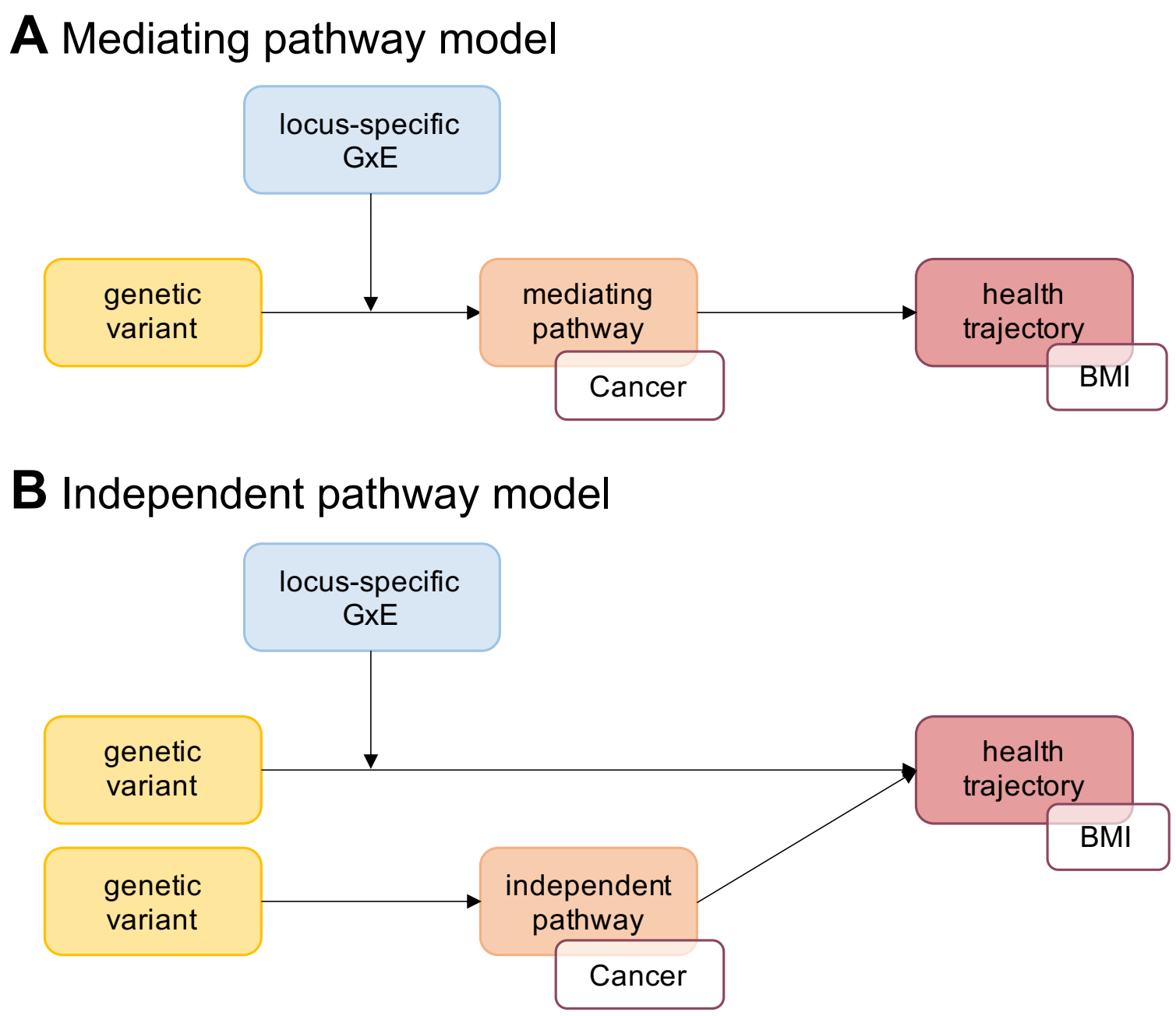

Supplementary Figure 5 illustrates scenarios under which major health events (e.g., cancer occurrence) may influence inference on age-varying genetic effects for a longitudinal continuous trait (e.g., changes in BMI).

(A) Mediating pathway model: A genetic variant influences the occurrence of a health event (e.g., cancer), which in turn alters the subsequent trajectory of the trait. In this scenario, the estimated genetic effect on the trait reflects the total effect of the variant, operating partly or fully through its risk on the health event. Conditioning on the intermediate event would induce bias in the genetic effect estimate, by blocking part of the causal pathway. Studies aiming to disentangle such mechanisms therefore require explicit mediation or joint longitudinal–time-to-event models.

(B) Independent pathway model: A genetic variant influences the longitudinal trait trajectory independently of a health event, while the event itself is influenced by separate genetic factors. Adjusting for the event does not induce bias and may increase statistical power, by removing noise in the outcome that is due to random variation.

Supplementary Figure 6. Quantifycation of regression dilution bias

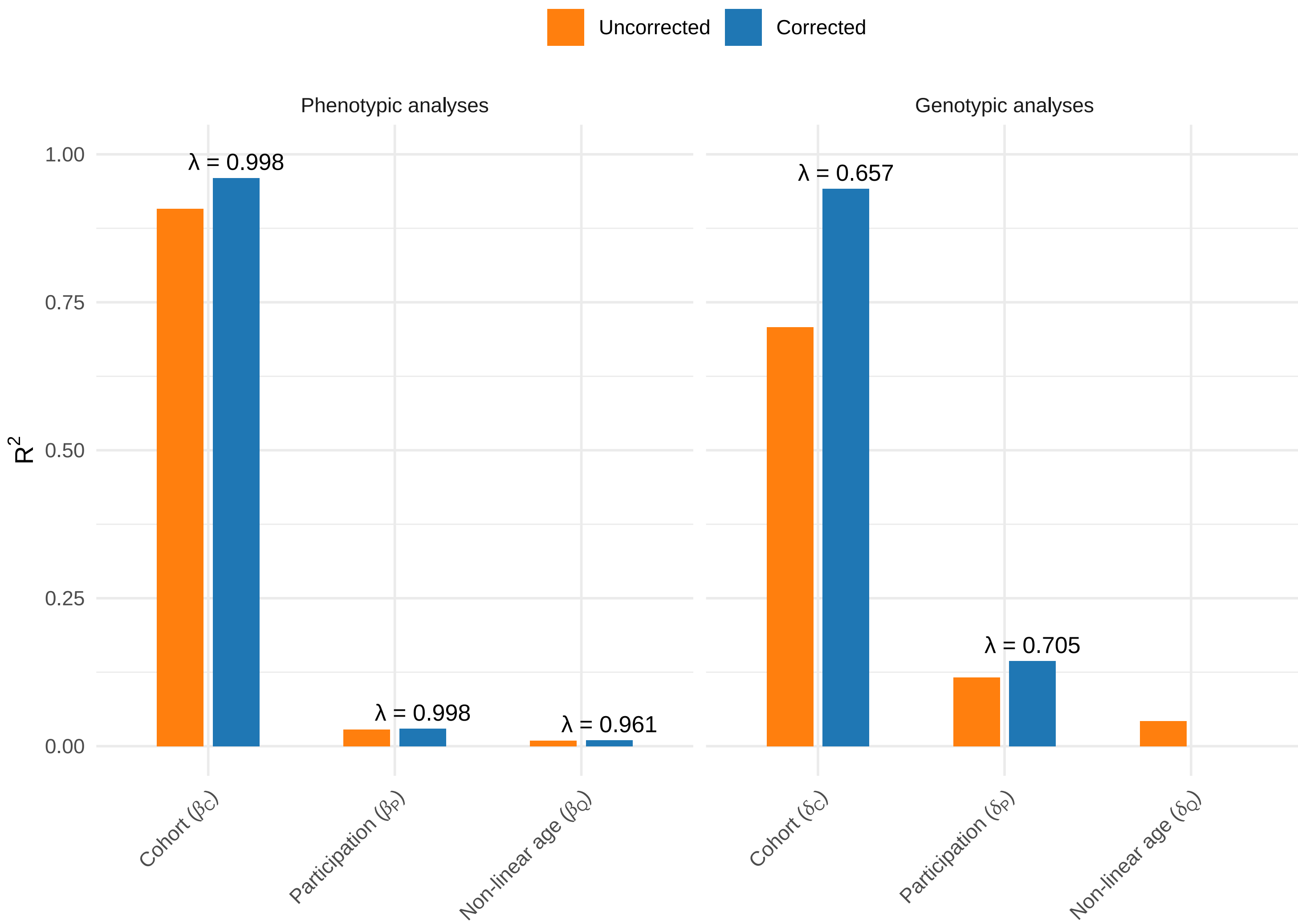

The figure shows the uncorrected (in in orange) and attenuation bias corrected (in in blue) R^2-contribution per factor (cohort effects, non-linear age effects, participation bias) to the observed effect differences between the cross-sectional and longitudinal design. As described and plotted in **Figure 2** and **Figure 5** in the main manuscript, the uncorrected R^2-contributions were obtained from two sets of analyses:

(1) Phenotypic analyses (**Figure 2**): $\beta_{\mathrm{L}} - \beta_{\mathrm{CS}} \sim \alpha_0 + \alpha_1 \cdot \beta_{\mathrm{C}} + \alpha_2 \cdot \beta_{\mathrm{Q}} + \alpha_3 \cdot \beta_{\mathrm{P}}$

(2) Genotypic analyses (**Figure 5**): $\delta_{\mathrm{L}} - \delta_{\mathrm{CS}} \sim \alpha_0 + \alpha_1 \cdot \delta_{\mathrm{C}} + \alpha_2 \cdot \delta_{\mathrm{Q}} + \alpha_3 \cdot \delta_{\mathrm{P}}$

We corrected for attenuation bias by dividing the observed variance components by a correction factor λ, such that $R^2_{\mathrm{corrected}} = R^2_{\mathrm{observed}}/\lambda$. Here, λ is the dilution ratio ($\lambda \in [0, 1]$), which quantifies the proportion of total observed variance in regression coefficients that reflects true underlying effects: $\lambda = \frac{V_{\mathrm{total}} - V_{\mathrm{error}}}{V_{\mathrm{total}}}$, with

$V_{\text{total}} = \frac{1}{n-1}\sum_{i=1}^{n}(\hat{\theta}_i - \mu)^2$ and $V_{\text{error}} = \frac{1}{n}\sum_{i=1}^{n} SE(\hat{\theta}_i)^2$. $\hat{\theta}$ denotes the vector of estimated coefficients for a given factor (e.g., $\hat{\theta}_i = \hat{\beta}_{\text{C}_i}$ for cohort effects) and $\mu = \frac{1}{n}\sum_{i=1}^{n}\hat{\theta}_i$. Lower values of λ indicate greater measurement error and stronger attenuation bias. The corrected R^2-estimates were rescaled to sum up to 1.

Of note, participation effects were defined as the difference between estimates obtained from a representative (weighted, W) and a less representative (unweighted, U) sample ($\hat{\theta}_{\text{P}} = \hat{\theta}_{\text{W}} - \hat{\theta}_{\text{U}}$). The corresponding standard errors were obtained as: $SE(\hat{\theta}_{\text{P}}) = \sqrt{SE(\hat{\theta}_{\text{W}})^2 + SE(\hat{\theta}_{\text{U}})^2 - 2r \cdot SE(\hat{\theta}_{\text{W}}) \cdot SE(\hat{\theta}_{\text{U}})}$, where $\hat{\theta} \in \hat{\beta}, \hat{\delta}$ represents either phenotypic or genotypic estimates. To obtain r, we performed simulations using data from 80,000 UK Biobank participants with available sampling weights and taking part in follow-up assessments. In 1,000 iterations, we generated normally distributed variables X and Y, and fit two linear models per iteration: (1) a weighted regression using normalized sampling weights, and (2) an unweighted regression with all weights set to 1. The correlation between the regression coefficients obtained from $Y \sim X$ across iterations was used to approximate r. This yielded an estimated value of $r = 0.6$.

Supplementary Figure 7. Implications for polygenic score approaches

To assess whether the differences observed between cross-sectional and longitudinal modeling approaches also extend to polygenic effects, we re-estimated models B1–B5 by replacing single-variant predictors (G_j) with trait-specific polygenic scores (PGS). We then compared the resulting age-varying polygenic effects across models. **Supplementary Figure 7** presents the PGS associations estimated from the five modeling approaches.

The PGS were constructed using PRSice-2[4], based on summary statistics derived from an independent discovery sample of the UK Biobank, generated using REGENIE[5] . The discovery sample included up to 322,213 participants who did not take part in longitudinal assessments. In PRSice-2, we included all LD-independent variants (–pval 1 —clump-kb 10000 –clump-r2 0.001), and adjusted for population structure using the first 20 principal components (–covar PC1–PC20).

The resulting PGS were evaluated in an independent target sample comprising up to 69,246 unrelated individuals of European ancestry participating in the longitudinal assessments. The variance explained (R^2) by each PGS for each phenotype, along with trait-specific discovery sample sizes from the genome-wide analyses, is reported in **Supplementary Data 4**.

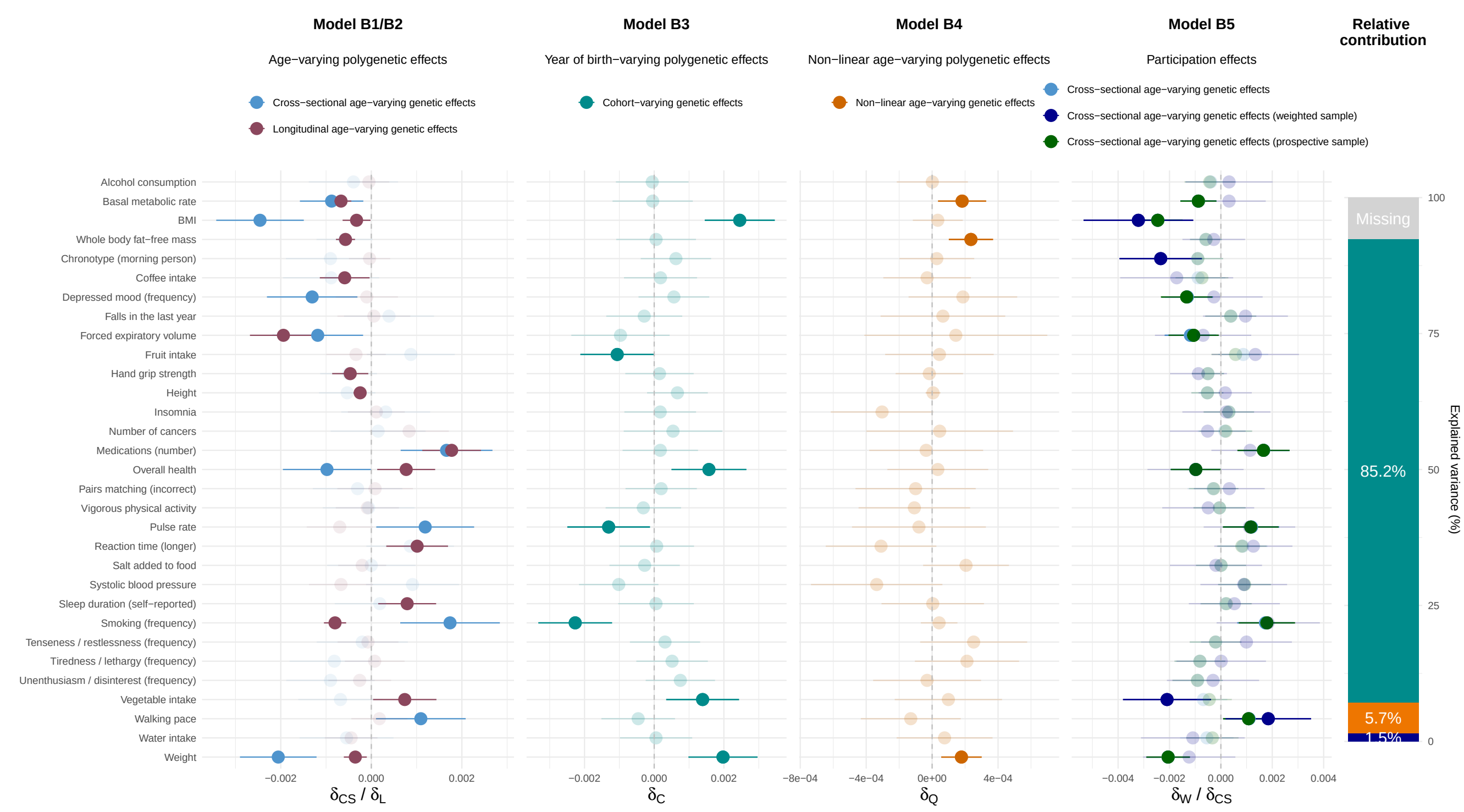

Supplementary Figure 8. Implications for Mendelian Randomization analyses

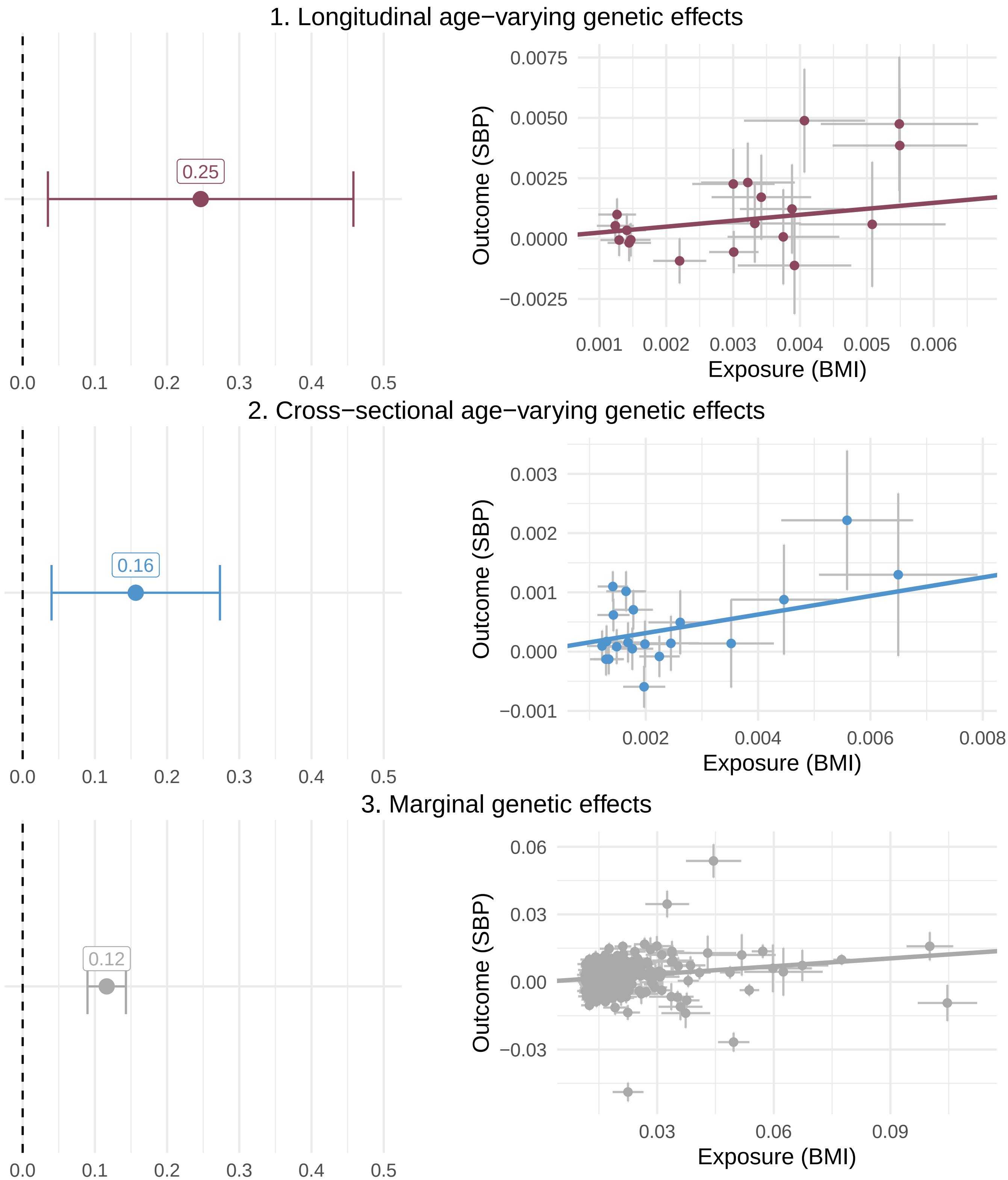

Supplementary Figure 8 shows the results obtained from Mendelian Randomization (MR) analyses, estimating the causal effect of body mass index (BMI) on systolic blood pressure (SBP), a well-established

causal effect we used as a positive control and proof-of-principle to illustrate the potential application of a longitudinal MR framework.

We performed standard two-sample MR using SNP effects derived from (i) marginal genome-wide models, and (ii) age-varying genetic effects estimated from cross-sectional and longitudinal interaction models. Causal effects were estimated using the inverse-variance weighted (IVW) estimator as implemented in the R package TwoSampleMR[6]. For the marginal model, LD-independent SNPs were selected using clumping parameters (–clump-kb 10,000; –clump-r^2 0.001), and genome-wide significant variants ($P < 5\times^{10-8}$) were used as instruments for BMI. For the age-varying models, the number of Bonferroni-significant SNPs was too small to conduct adequately powered MR analyses (k = 3 from the cross-sectional model; k = 2 from the longitudinal model, c.f., **Figure 3A** in the main text). Therefore, for illustrative purposes only, we selected the top 20 LD-independent variants ranked by statistical significance from each of the two genome-wide analyses. We emphasize that this does not constitute a valid instrument selection strategy and is presented solely to demonstrate the potential application of longitudinal MR. As such, the resulting causal estimates should be interpreted with caution.

The forest plots on the left display the causal effect with corresponding 95% confidence interval of the exposure (BMI) on the outcome (SBP). The scatter plots on the right show the instrument-specific effect estimates with corresponding 95% confidence intervals, with the fitted regression line indicating the inverse variance weighted estimated causal effect.

Supplementary note on change ratios

In our two-step genome-wide approach to detect age-varying genetic effects, we used the annualized rate of phenotypic change as the outcome. This was defined as $P_{\Delta_j} = \frac{P_{1j} - P_{0j}}{FU_j}$, where P_{0j} and P_{1j} denote the baseline and most recent follow-up measurement for individual and FU_j is the corresponding follow-up duration. Although this formulation is computationally efficient for large-scale genome-wide analyses, it relies on several assumptions that warrant consideration.

First, because $\frac{P_{1j}-P_{0j}}{FU_j} = \mathbf{W}\cdot\alpha + \delta_{\mathrm{L}}\cdot G_j + \varepsilon_j$ can be rearranged to $P_{1j} - P_{0j} = \alpha(FU_j\cdot\mathbf{W}) + \delta_{\mathrm{L}}(FU_j\cdot G_j) + FU_j\cdot\varepsilon_j$, the ratio formulation implicitly implies that change increases linearly with follow-up time, that the SNP effect on change scales linearly with follow-up duration, and the variance of the change score is proportional to the follow-up time squared – assumptions that are not directly testable within a simple linear regression framework. However, given current sample sizes and realistic interaction effects, detecting age-specific effects other than linearly increasing or decreasing would be severely underpowered in most genome-wide settings. In this context, the assumption of heteroscedastic error may also be reasonable, as many other factors influencing complex traits are likely to operate in an age-dependent manner, consistent with empirical studies showing that the variance of several complex traits increases with age (e.g., BMI[7], SBP[8]).

Second, ratio outcomes can be sensitive to associations with the denominator. If a genetic variant influences follow-up duration, dividing by P_{Δ_j} may induce an association between genotype and the change outcome even in the absence of a direct genetic effect on phenotypic change[9,10]. As a sensitivity analysis, this possibility can be evaluated empirically, for example by conducting genome-wide association analyses with follow-up duration (FU_j) as the outcome.

Given these limitations of difference-score approaches, mixed-effects models are generally preferred for studying age-varying genetic effects[11–13]. Compared to simple change models, mixed-effects models have been shown to better control false positives, yield more precise parameter estimates, and reduce bias in gene-by-age interaction effects in simulation studies[11]. In addition, their flexibility allows explicit evaluation of modeling assumptions, including non-linear age effects as examined here (c.f., Models A4/B4 in the main text). Although computationally more demanding, recent software developments such as GMMAT/MAGEE[13] are now making genome-wide applications of mixed-effects models increasingly feasible.

References

1. Schoeler, T. *et al.* Participation bias in the UK biobank distorts genetic associations and downstream analyses. *Nature Human Behaviour* **7**, 1216–1227 (2023).
2. Lumley, T. *Complex surveys: A guide to analysis using r.* (John Wiley & Sons, 2011).
3. Lumley, T. & Huang, X. Linear mixed models for complex survey data: Implementing and evaluating pairwise likelihood. *Stat* **13**, e657 (2024).
4. Choi, S. W. & O'Reilly, P. F. PRSice-2: Polygenic risk score software for biobank-scale data. *Gigascience* **8**, giz082 (2019).
5. Mbatchou, J. *et al.* Computationally efficient whole-genome regression for quantitative and binary traits. *Nature genetics* **53**, 1097–1103 (2021).
6. Hemani, G. *et al.* The MR-base platform supports systematic causal inference across the human phenome. *elife* **7**, e34408 (2018).
7. Silventoinen, K. *et al.* Differences in genetic and environmental variation in adult BMI by sex, age, time period, and region: An individual-based pooled analysis of 40 twin cohorts. *The American journal of clinical nutrition* **106**, 457–466 (2017).
8. Satoh, M. *et al.* Age-related trends in home blood pressure, home pulse rate, and day-to-day blood pressure and pulse rate variability based on longitudinal cohort data: The ohasama study. *Journal of the American Heart Association* **8**, e012121 (2019).
9. Mooldijk, S. S., Labrecque, J. A., Ikram, M. A. & Ikram, M. K. Ratios in regression analyses with causal questions. *American Journal of Epidemiology* **194**, 311–313 (2025).
10. McCaw, Z. R. *et al.* Pitfalls in performing genome-wide association studies on ratio traits. *Human Genetics and Genomics Advances* **6**, (2025).
11. Wiegrebe, S. *et al.* Analyzing longitudinal trait trajectories using GWAS identifies genetic variants for kidney function decline. *Nature communications* **15**, 10061 (2024).
12. Ko, S. *et al.* GWAS of longitudinal trajectories at biobank scale. *The American Journal of Human Genetics* **109**, 433–445 (2022).
13. Zhang, M. *et al.* Robust mixed model association test for gene-environment interactions. *medRxiv* (2025) doi:10.1101/2025.10.01.25336808.
