# Supplementary material for: Design and model choices shape inference of age-varying genetic effects on complex traits
Source: Nat Aging. 2026 Oct 6;6(10):2253–64. doi: 10.1038/s43587-026-01232-w (PMC13661259; doi:10.1038/s43587-026-01232-w)
Supplement: Supplementary file 3 [file 43587_2026_1232_MOESM3_ESM.pdf]

nature portfolio

Peer Review File

Design and model choices shape inference of age-varying genetic effects on complex traits

Corresponding Author: Dr Tabea Schoeler

This file contains all reviewer reports in order by version, followed by all author rebuttals in order by version.

Attachments originally included by the reviewers as part of their assessment can be found at the end of this file.

Version 0:

Reviewer comments:

Reviewer #1

(Remarks to the Author)
The authors explore the use of cross-sectional and longitudinal approaches to identify age-varying genetic effects in a subset of European ancestry UK Biobank participants. The analysis is well-thought out. Methods are well-described with clearly stated cross-sectional and longitudinal models, as well as an investigation into sources of discrepancy between their estimates of age-varying genetic effects, appropriately accounting for multiple testing. Strengths and limitations of both approaches are clearly explained, and the way they complement each other is effectively illustrated.

Thinking forward, my only comment is for the Discussion:

- The analysis focuses on variants with MAF>0.01. In the Discussion, it would be interesting for the authors to discuss the possibility of extensions to rare or low-frequency variants.

(Remarks on code availability)
I was unable to review the code as an error message for the link was given. However, this does not concern me as the analyses rely on standard software.

Reviewer #2

(Remarks to the Author)
This is an interesting methodological paper investigating alternative design choices to explore gene by age interactions. While a number of SNP-by-age interactions are identified, the authors did not make a clear case how is this going to be useful for future studies like PRS development MR analysis or gaining biological insights. Without showing downstream implications, this seems like an interesting technical excercise without clear applications.

Major comments

1) The authors may want to study if effects of existing polygenic scores for some of these traits varies by age using alternative approaches. As many PGS are getting closer to clinical applications, there could be some implications for age-dependent effects.

2) One challenge for longitudinal analysis of various traits over substantial time span is how to account for major events, e.g cancer occurrence, which may substantially alter the longitudinal process (e.g BMI trajectory) and themselves have genetic components. Ideally, models should be set up to understand how genetic effects on a trait may change over time before such events happen. This is a complex problem and requires joint modeling of longitudinal data and time-to-event outcomes. This issue was not addressed or discussed.

3) I wonder whether the authors can show some implications for MR analysis using SNPs which show age-dependent effects. Could these be used to show change in some trait values, e.g BMI, are causally associated with disease outcome.

(Remarks on code availability)

Reviewer #3

(Remarks to the Author)
Please see the attached PDF.

(Remarks on code availability)
I was not able to run the code, though in general it appears to be well-documented. I would suggest the authors apply tidyverse styling to their R code.

Version 1:

Reviewer comments:

Reviewer #1

(Remarks to the Author)
My comments have been addressed.

(Remarks on code availability)
The scripts are well organised and clearly annotated.

Reviewer #3

(Remarks to the Author)
The authors have thoughtfully and substantively addressed all of my comments. I congratulate them on their work.

(Remarks on code availability)

Open Access This Peer Review File is licensed under a Creative Commons Attribution 4.0 International License, which permits use, sharing, adaptation, distribution and reproduction in any medium or format, as long as you give appropriate credit to the original author(s) and the source, provide a link to the Creative Commons license, and indicate if changes were made.
In cases where reviewers are anonymous, credit should be given to 'Anonymous Referee' and the source.
The images or other third party material in this Peer Review File are included in the article's Creative Commons license, unless indicated otherwise in a credit line to the material. If material is not included in the article's Creative Commons license and your intended use is not permitted by statutory regulation or exceeds the permitted use, you will need to obtain permission directly from the copyright holder.
To view a copy of this license, visit https://creativecommons.org/licenses/by/4.0/

We thank the reviewers for their thorough and constructive feedback. In response, we have extended the manuscript to include (1) new analyses illustrating implications for downstream applications, including polygenic scores and Mendelian randomization; (2) additional sensitivity analyses addressing longitudinal attrition; and (3) expanded discussion clarifying key assumptions and conceptual considerations underlying our analytical choices.

Please note that our responses are highlighted in blue and all changes to the manuscript are highlighted in green.

Reviewer 1

The authors explore the use of cross-sectional and longitudinal approaches to identify age-varying genetic effects in a subset of European ancestry UK Biobank participants. The analysis is well-thought out. Methods are well-described with clearly stated cross-sectional and longitudinal models, as well as an investigation into sources of discrepancy between their estimates of age-varying genetic effects, appropriately accounting for multiple testing. Strengths and limitations of both approaches are clearly explained, and the way they complement each other is effectively illustrated. Thinking forward, my only comment is for the Discussion:

R1.1: The analysis focuses on variants with MAF>0.01. In the Discussion, it would be interesting for the authors to discuss the possibility of extensions to rare or low-frequency variants.

We thank the reviewer for the positive feedback on our work. If rare genetic variants impacting longitudinal outcomes behave similarly to common variants (i.e., explained variance increases with MAF), we expect that analyses testing rare variants will have less power and hence larger sample sizes are needed to detect such effects. We do agree, however, that increasing sample sizes with WGS data will enable to test these in the future. The discussion has now been extended as follows:

> [Manuscript, Discussions]: "With the growing availability of whole-genome sequencing and longitudinal data (e.g., electronic health records), future studies will also be able to extend these analyses to low-frequency and rare variants, potentially uncovering additional age-varying genetic effects."

Remarks on code availability: I was unable to review the code as an error message for the link was given. However, this does not concern me as the analyses rely on standard software.

We apologize for this glitch. While all analytical code was available in the analysis folder of the corresponding GitHub repository, some of the links to these files in the README markdown document were outdated. This has now been corrected.

Reviewer 2

This is an interesting methodological paper investigating alternative design choices to explore gene by age interactions. While a number of SNP-by-age interactions are identified, the authors did not make a clear case how is this going to be useful for future studies like PRS development MR analysis or gaining biological insights. Without showing downstream implications, this seems like an interesting technical excercise without clear applications.

We agree that clarifying the relevance of our findings for downstream applications would be a valuable addition to the manuscript. In response, we have conducted the proposed analyses, including polygenic score and Mendelian Randomization analysis, and have expanded the Discussion accordingly in the revised version.

R2.1: The authors may want to study if effects of existing polygenic scores for some of these traits varies by age using alternative approaches. As many PGS are getting closer to clinical applications, there could be some implications for age-dependent effects.

We agree that this is an important consideration. To address this point, we have now extended our analytical framework to polygenic scores. Specifically, we conducted genome-wide association analyses for the 31 included traits in an independent subset of the UK Biobank and constructed trait-specific PGS in a separate, independent target sample. We then re-estimated models B1–B5 by replacing single-variant predictors with the trait-specific PGSs. These additions are now described in the revised manuscript:

> [Manuscript, Methods]: "Further, to evaluate the implications of cross-sectional versus longitudinal modeling for polygenic score (PGS) applications, we re-estimated models B1–B5 replacing single-variant predictors (Gj) with trait-specific polygenic scores. We then compared the resulting age-varying polygenic effects across the different models (see **Supplementary Note** for details)."
>
> [Manuscript, Results]: "When fitting the same set of models to polygenic effects, the findings mirrored those observed at the SNP level. Specifically, PGS-by-gene effects were identified for anthropometric traits and metabolic indicators (BMI, weight, and basal metabolic rate), with consistent directions of association but variation in effect magnitudes. When examining predictors of these discrepancies, cohort confounding emerged as the most relevant contributor, explaining 85.2% of the observed differences (**Supplementary Figure 5**). Together, these results highlight that, for certain traits, PGS performance varies across contexts. This underscores the importance of taking into account both individual-level characteristics and social factors when evaluating the applicability and generalizability of PGS."
>
> [Supplement Figure 5]: "To assess whether the differences observed between cross-sectional and longitudinal modeling approaches also extend to polygenic effects, we re-estimated models B1–B5 by replacing single-variant predictors (G_j) with trait-specific polygenic scores (PGS). We then compared the resulting age-varying polygenic effects across models. **Supplementary Figure 5** presents the PGS associations estimated from the five modeling approaches.

The PGS were constructed using PRSice-2[4], based on summary statistics derived from an independent discovery sample of the UK Biobank, generated using REGENIE[5] . The discovery sample included up to 322,213 participants who did not take part in longitudinal assessments. In PRSice-2, we included all LD-independent variants (–pval 1 –-clump-kb 10000 –clump-r2 0.001), and adjusted for population structure using the first 20 principal components (–covar PC1–PC20).

The resulting PGS were evaluated in an independent target sample comprising up to 69,246 unrelated individuals of European ancestry participating in the longitudinal assessments. The variance explained (R^2) by each PGS for each phenotype, along with trait-specific discovery sample sizes from the genome-wide analyses, is reported in **Supplementary Data 4**.”

Supplementary Figure 5.

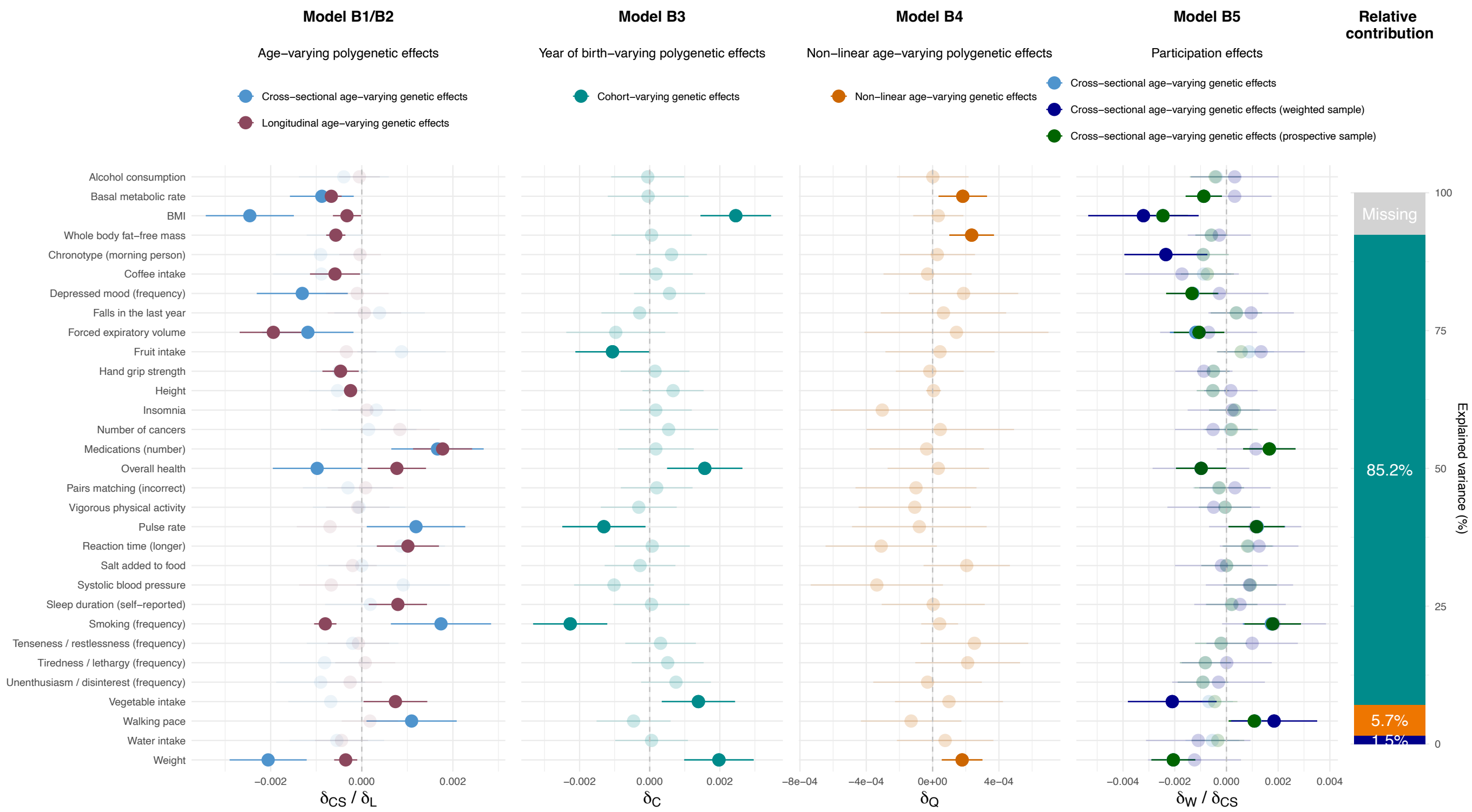

R2.2: One challenge for longitudinal analysis of various traits over substantial time span is how to account for major events, e.g cancer occurrence, which may substantially alter the longitudinal process (e.g BMI trajectory) and themselves have genetic components. Ideally, models should be set up to understand how genetic effects on a trait may change over time before such events happen. This is a complex problem and requires joint modeling of longitudinal data and time-to-event outcomes. This issue was not addressed or discussed.

We thank the reviewer for raising this important point. As pointed out, major events such as disease onset (e.g., cancer), which may themselves have a genetic basis, can substantially alter longitudinal health trajectories and complicate the mechanistic understanding of age-varying genetic effects. To clarify how such events may influence inference in longitudinal genetic analyses, we have expanded the Discussion and Supplement to outline two conceptual scenarios through which third factors can affect observed trajectories.

[Manuscript, Discussion]: "Finally, it is important to note that the identified age-varying genetic effects may operate through indirect pathways, and a deeper understanding of these mechanisms will require more tailored modeling approaches. For example, longitudinal trajectories can be substantially altered by major health events occurring during follow-up, such as disease onset. When such events are themselves genetically influenced, explicitly modeling them can facilitate more precise mechanistic interpretations. As detailed in **Supplementary Figure 8**, this includes (i) mediating pathways, in which genetic effects on a trait operate through an intermediate event, and (ii) independent pathways, in which genetic effects on the event and the trait act separately."

[Supplementary Figure 8]:

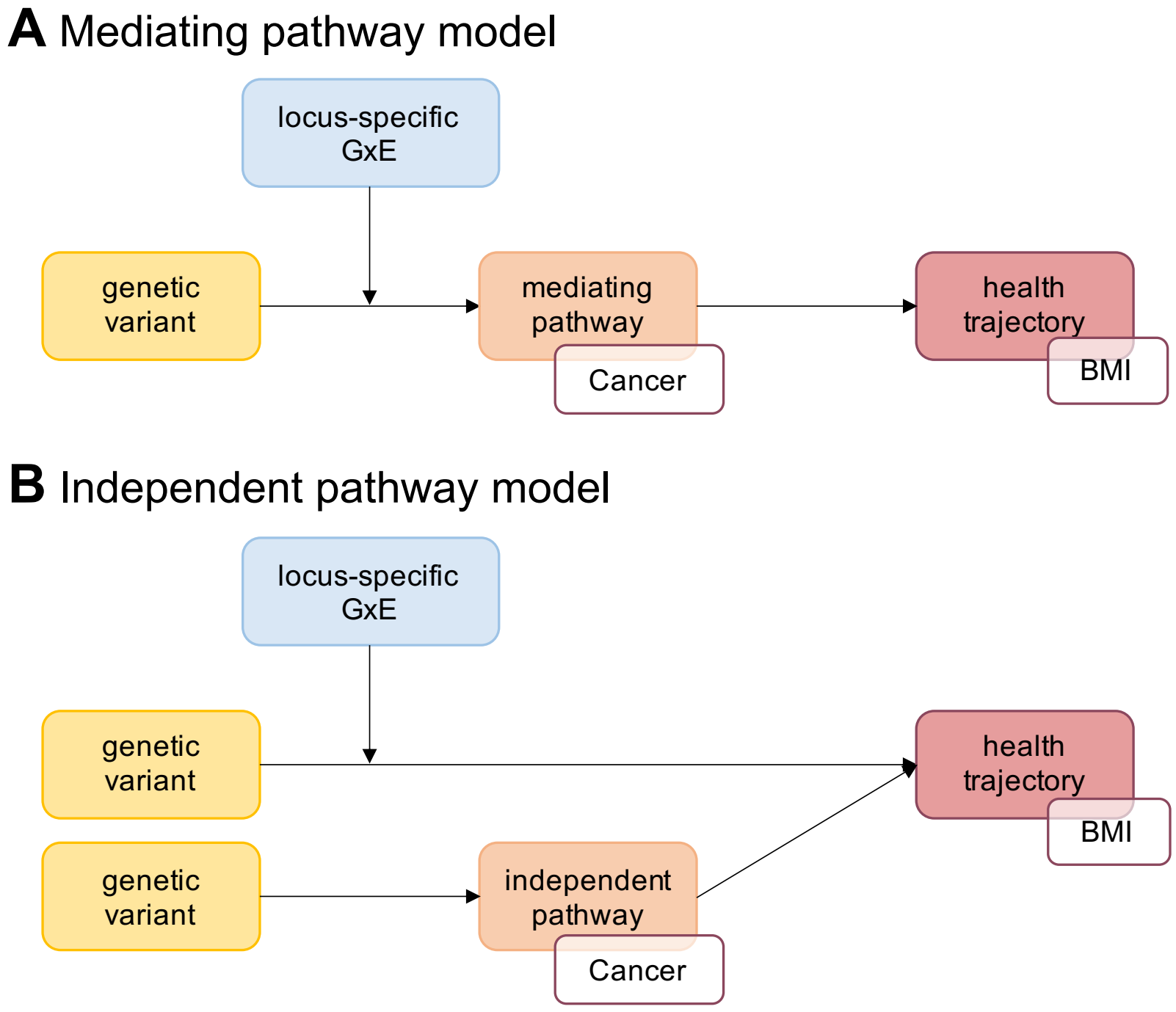

Supplementary Figure 8 illustrates scenarios under which major health events (e.g., cancer occurrence) may influence inference on age-varying genetic effects for a longitudinal continuous trait (e.g., changes in BMI).

(A) Mediating pathway model: A genetic variant influences the occurrence of a health event (e.g., cancer), which in turn alters the subsequent trajectory of the trait. In this scenario, the estimated genetic effect on the trait reflects the total effect of the variant, operating partly or fully through its risk on the health event. Conditioning on the intermediate event would induce bias in the genetic effect estimate, by blocking part of the causal pathway. Studies aiming to disentangle such

mechanisms therefore require explicit mediation or joint longitudinal–time-to-event models.

(B) Independent pathway model: A genetic variant influences the longitudinal trait trajectory independently of a health event, while the event itself is influenced by separate genetic factors. Adjusting for the event does not induce bias and may increase statistical power, by removing noise in the outcome that is due to random variation.

R2.3: I wonder whether the authors can show some implications for MR analysis using SNPs which show age-dependent effects. Could these be used to show change in some trait values, e.g BMI, are causally associated with disease outcome.

We thank the reviewer for this interesting suggestion. We have now expanded the manuscript to discuss the potential utility of longitudinal Mendelian Randomization (MR) and included an illustrative analysis applying such a framework. Specifically, we examined whether age-varying genetic effects for BMI can be used as instruments to identify the causal effect of BMI on systolic blood pressure (SBP), a well-established causal relationship used here as a positive control.

> [Manuscript, Methods]: "Finally, we illustrate the implications of age-varying genetic effects for causal effect estimation using Mendelian randomization (MR), a widely applied method that leverages genetic instruments to estimate the causal effect of an exposure on an outcome[43]. While most MR studies rely on genome-wide association estimates derived from time-invariant models, there are several reasons why a longitudinal MR framework may be informative. First, standard MR estimates are typically interpreted as reflecting lifelong genetic predisposition to higher exposure levels[44], rather than the effect of modifying the exposure over a defined period. A longitudinal framework, estimating the effect of annual change in the exposure on annual change in the outcome, may therefore offer an interpretation more closely aligned with intervention effects. Second, genetic influences on mean levels and on longitudinal change have been shown to be mostly distinct[18]. Consequently, potential violations of MR assumptions, such as horizontal pleiotropy or confounding, are likely to differ between time-invariant and time-varying genetic instruments. Triangulating across these approaches may therefore strengthen causal inference. Details on the longitudinal MR approach employed here can be found in the **Supplementary Note**."

> [Manuscript, Results]: "Finally, we illustrate the potential utility of age-varying genetic effects for causal inference within a Mendelian randomization (MR) framework, by examining whether longitudinal MR can recover the well-established adverse effect of BMI on systolic blood pressure (SBP). As shown in **Supplementary Figure 6**, the estimated effects were directionally consistent with the known positive causal relationship between BMI and SBP when using age-varying genetic instruments. However, estimates from the longitudinal MR approach came with substantially greater uncertainty, due to the small number of available instruments and the weaker and noisier SNP-exposure associations.

[Manuscript, Discussion]: "Finally, we explore the implications of age-varying genetic effects for causal inference using Mendelian randomization (MR). Although presented for illustration only, these analyses demonstrate how longitudinally derived genetic effects could, in principle, be incorporated into MR frameworks to test if changes in an exposure translate into changes in an outcome. In practice, adequately powered longitudinal MR will require substantially larger genome-wide association studies to identify a sufficient number of strong instruments indexing change. Moreover, longitudinal MR estimates are only interpretable under additional assumptions, namely that time-varying genetic effects are independent of lifetime genetic effects and that selected instruments do not exhibit higher-order age-dependent effects (e.g., gene-by-age^2 interactions). Evaluating these (testable) assumptions should therefore be an integral part of sensitivity analyses when applying this approach."

[Supplement Figure 8]: "Supplementary Figure 6. Mendelian Randomization analyses"

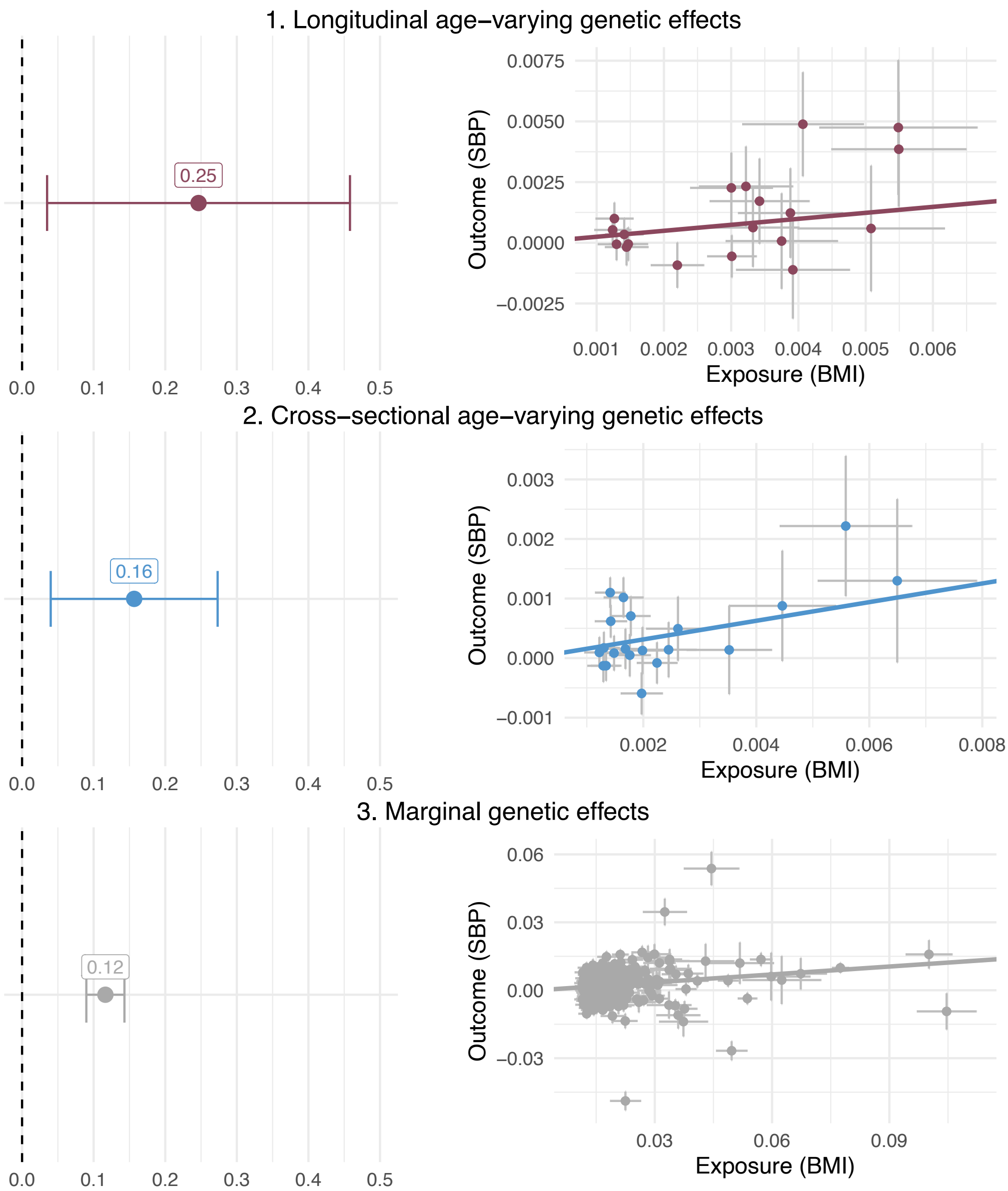

Supplementary Figure 6 shows the results obtained from Mendelian Randomization (MR) analyses, estimating the causal effect of body mass index (BMI) on systolic blood pressure (SBP), a well-established causal effect we used as a positive control and proof-of-principle to illustrate the potential application of a longitudinal MR framework.

We performed standard two-sample MR using SNP effects derived from (i) marginal genome-wide models, and (ii) age-varying genetic effects estimated from cross-sectional and longitudinal interaction models. Causal effects were estimated using the inverse-variance weighted (IVW) estimator as implemented in the R package TwoSampleMR[6]. For the marginal model, LD-independent SNPs were selected using clumping parameters (–clump-kb 10,000; –clump-r^2 0.001), and genome-wide significant variants ($P < 5 \times^{10-8}$) were used as instruments for BMI. For the age-varying models, the number of Bonferroni-significant SNPs was too small to conduct adequately powered MR analyses (k = 3 from the cross-sectional model; k = 2 from the longitudinal model, c.f., **Figure 3A** in the main text). Therefore, for illustrative purposes only, we selected the top 20 LD-independent variants ranked by statistical significance from each of the two genome-wide analyses. We emphasize that this does not constitute a valid instrument selection strategy and is presented solely to demonstrate the potential application of longitudinal MR. As such, the resulting causal estimates should be interpreted with caution.

The forest plots on the left display the causal effect with corresponding 95% confidence interval of the exposure (BMI) on the outcome (SBP). The scatter plots on the right show the instrument-specific effect estimates with corresponding 95% confidence intervals, with the fitted regression line indicating the inverse variance weighted estimated causal effect.

Reviewer 3

This manuscript addresses the distinction between cross-sectional and longitudinal age-varying genetic effects, an important topic where confusion abounds. The approach is rigorous, the exposition is clear, and the conclusion that significant cohort effects exist for many of the traits studied is scientifically significant. My main suggestion relates to the manuscript's current framing. In particular, the current exposition implicitly assumes that the cross-sectional and longitudinal models target the same estimand. In general, they do not. To avoid perpetuating this misconception, please state clearly and early on that these analyses target different estimands and, consequently, their estimates need not coincide (in general). Only under strong assumptions, which the manuscript should make explicit (e.g., no cohort- or period-by-genotype effect modification), are estimates from the two models expected to agree. Absent this context, describing the cross-sectional estimates as "biased" relative to the longitudinal estimates conflates estimator bias with estimand mismatch.

We thank the reviewer for this important comment. We fully agree that cross-sectional and longitudinal models do not necessary target the same estimand. However, our study was motivated by the observation that age-varying genetic effects are commonly quantified using cross-sectional interaction models (c.f., examples shown in the table below), likely due to the unavailability of sufficiently large longitudinal dataset. Cross-sectional analyses do not have a clear estimand as such estimators pick up a mixture of multiple phenomenon – thus they are not performed with the specific aim to estimate a mixture of multiple interesting parameters. Based on our literature search (see below), the most frequent framing of their estimand is the detection of age-dependent genetic effects, i.e., their estimands are not accurately framed.

With the increasing availability of repeated measures in biobanks, longitudinal approaches are now more frequently applied to study age-dependent genetic effects. As a result, in much of the literature, findings from both designs are interpreted as addressing the same scientific question, that is, how genetic effects vary with age. We therefore considered it important to systematically evaluate how estimates from the two designs compare and to clarify the conditions under which they agree or disagree. We have now revised the introduction to make this clearer, and have also highlighted that agreement between cross-sectional and longitudinal analyses relies on strong assumptions. In addition, we have revised the manuscript to avoid characterizing discrepancies between designs as "bias".

> [Manuscript, Introduction]: "Both cross-sectional and longitudinal approaches have been used to study age-varying genetic effects and related downstream processes[10–14,18–21], often implicitly assuming that they target the same quantity of interest (that is, the same estimand). However, agreement between the two designs requires strong assumptions, including the absence of cohort- or period-by-genotype effect modification, alongside comparable sampling and study participation characteristics."

Examples of **cross-sectional studies** focusing on **age-varying genetic effects** as their estimand of interest

**Study**	**Study note**
Robinson, M.R., English, G., Moser, G., Lloyd-Jones, L.R., Triplett, M.A., Zhu, Z., Nolte, I.M., van Vliet-Ostaptchouk, J.V., Snieder, H., LifeLines Cohort Study and Esko, T., 2017. Genotype–covariate interaction effects and the heritability of adult body mass index. *Nature genetics*, *49*(8), pp.1174-1181.	"We divided the sample into five age groups, representative of different stages of adult life."
Winkler, T.W. et al. The influence of age and sex on genetic associations with adult body size and shape: a large-scale genome-wide interaction study. *PLoS Genet.* **11**, e1005378 (2015).	"To find age-different genetic effects, we computed age-difference P-values ($P_{agediff}$) by testing for difference between the age group-specific meta-analyzed beta-estimates"
Namba, S., Sonehara, K., Koyanagi, Y.N., Kikuchi, T., Ojima, T., Edahiro, R., Sato, G., Yamaji, T., Tomofuji, Y., Ueda, H. and Yamamoto, K., 2026. A cross-population compendium of gene–environment interactions. *Nature*, pp.1-10.	"We divided BBJ1 and UKB1 into two equal-sized age groups and conducted GWAS for pulse pressure within each group."
Labrecque, Jeremy A., and Sonja A. Swanson. "Age-varying genetic associations and implications for bias in Mendelian randomization." *MedRxiv* (2021): 2021-04.	"we are using cross-sectional data to infer individual-level trajectories over age"
Ao, L. *et al.* Large-scale genome-wide interaction analyses on multiple cardiometabolic risk factors to identify age-specific genetic risk factors. *GeroScience* 1–13 (2024).	"the GWIS [Genome-wide interaction analysis] of each cardiovascular risk factor was carried out for the included 270,276 UKB individuals by the generalized linear model, with covariates including age, sex, first ten genetic principal components (PCs) and an interaction term between genetic variant and age"
Gillespie, N.A., Gentry, A.E., Kirkpatrick, R.M., Reynolds, C.A., Mathur, R., Kendler, K.S., Maes, H.H., Webb, B.T. and Peterson, R.E., 2022. Determining the stability of genome-wide factors in BMI between ages 40 to 69 years. *PLoS Genetics*, *18*(8), p.e1010303.	"we performed BMI GWAS on six independent five-year age intervals between 40 and 72 years"
Leyden, G.M., Pagoni, P., Power, G.M., Carslake, D., Richardson, T.G., Tilling, K., Hemani, G., Smith, G.D. and Sanderson, E., 2025. An Evaluation of Age-varying Genetic Effects underlying Body-Mass Index and Blood Pressure in the UK Biobank. *medRxiv*, pp.2025-12.	"we conducted a series of age-stratified GWAS on individuals aged 40-69 years in the UK Biobank"
Chung, J.Y., Jung, H.U., Kim, D.J., Baek, E.J., Kim, H.K., Kang, J.O., Lim, J.E. and Oh, B., 2022. Identification of five genetic variants with differential effects on obesity-related traits based on age. *Frontiers in Genetics*, *13*, p.970657	"We conducted stratified analyses between young and old groups using genome-wide association studies"
Simino, J., Shi, G., Bis, J.C., Chasman, D.I., Ehret, G.B., Gu, X., Guo, X., Hwang, S.J., Sijbrands, E., Smith, A.V. and Verwoert, G.C., 2014. Gene-age interactions in blood pressure regulation: a large-scale investigation with the CHARGE, Global BPgen, and ICBP Consortia. *The American Journal of Human Genetics*, *95*(1), pp.24-38.	"We employed age stratification and metaregression to identify BP loci whose magnitude of genetic effects differ by age"
Winkler, T.W., Brandl, C., Grassmann, F., Gorski, M., Stark, K., Loss, J., Weber, B.H., Heid, I.M. and International Age-related Macular Degeneration Genomics Consortium (IAMDGC), 2018. Investigating the modulation of genetic effects on late AMD by age and sex: lessons learned and two additional loci. *PLoS One*, *13*(3), p.e0194321.	"We conducted age-group-stratified genome-wide association analyses"

R3.1: In the introduction, consider defining and contrasting cohort and age effects, and explaining why cohort effects can be mistaken for age effects without careful modeling.

We thank the reviewer for this suggestion and have revised the introduction as follows:

> [Manuscript, Introduction]: "Cohort effects reflect differences attributable to the historical and social contexts in which individuals were born and raised, such as shifts in educational systems, economic conditions, or public policies. Age effects, in contrast, refer to changes arising from the aging process itself as individuals grow older, such as declines in physiological function or cognitive performance. Because age and year of birth are inherently linked in cross-sectional data (age = period − cohort), between-cohort differences can be misattributed to aging if not explicitly accounted for. For example, cohort-varying genetic effects have been documented in response to societal changes, such as the collapse of the Soviet Union[22,23] or educational policy reforms[24] effects that would falsely be interpreted as age-varying genetic effects if tested cross-sectionally."

R3.2: Models (A5) and (B5) use inverse probability weighting to account for baseline participation. Was an analogous inverse-probability-of-censoring weighting analysis considered to address potentially informative dropout during follow-up? Relatedly, please estimate, if possible, the proportion of subjects lost to follow-up due to death, and please comment on how attrition due to death impacts the analysis.

We thank the reviewer for this suggestion. We have now generated sampling weights for participation in longitudinal assessments and examined how re-weighting affects the estimated age effects. We additionally quantified loss to follow-up due to death and comment on its potential implications below. The extended results and discussion now read as follows:

> [Results, Manuscript]: "Because selective dropout may influence longitudinal age estimates, we also derived inverse probability weights for participation in follow-up assessments (see Supplement). As shown in **Supplementary Figure 2**, re-weighting the follow-up sample to improve representativeness had, overall, a smaller impact on longitudinal age estimates than the impact observed following re-weighting of the cross-sectional sample to account for selective baseline participation. Of note, of the 399,661 participants not taking part in follow up research, 42,001 (11%) died prior to the first longitudinal assessment. Since the majority of loss to follow-up was due to reasons other than death, the observed changes in effect estimates likely reflect selective dropout mechanisms weakly related to mortality."

> [Results, Supplement]: "**Supplementary Figure 2. Correction for selective participation**

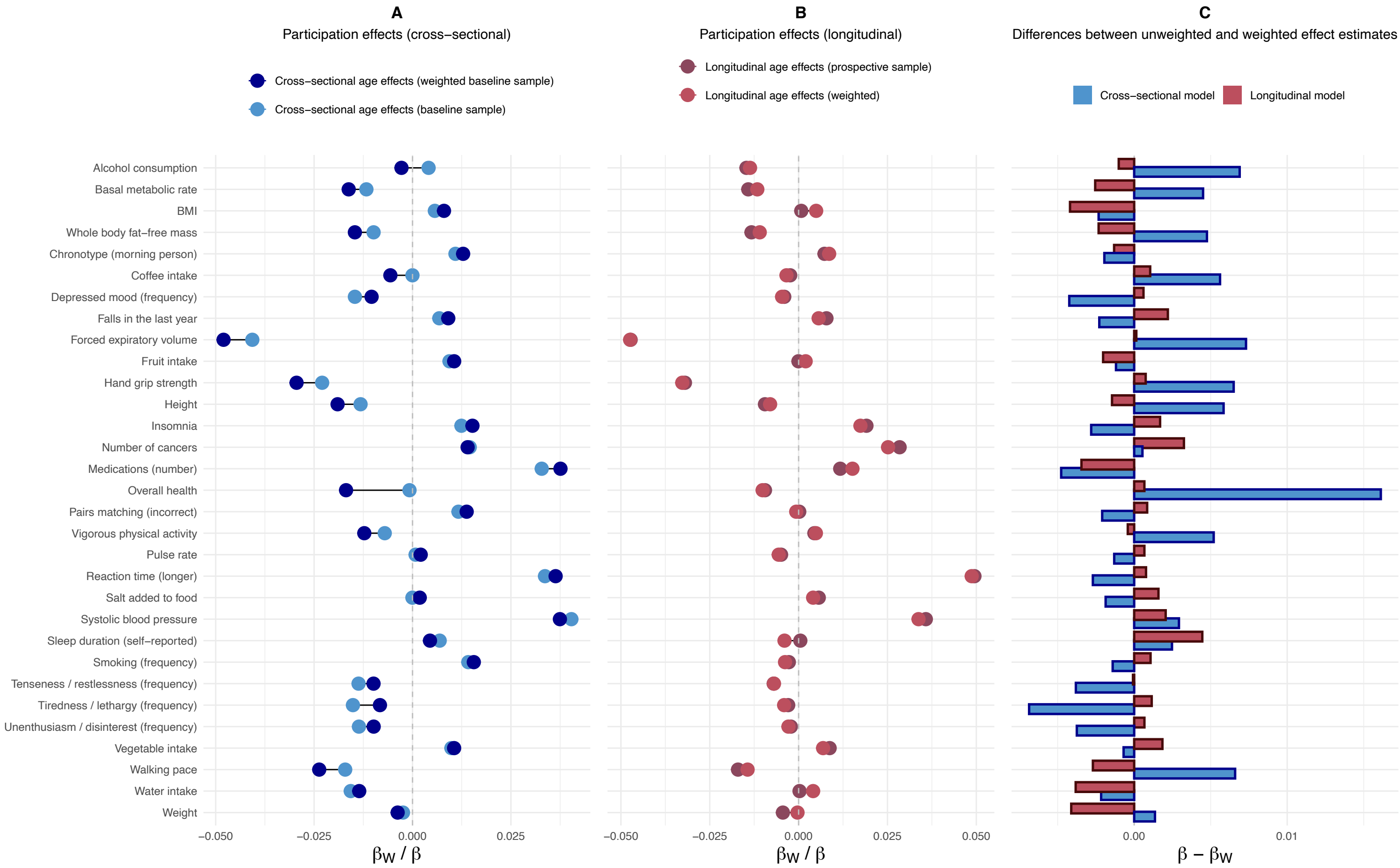

To assess the impact of selective participation, including selective baseline participation and selective follow-up participation, we applied inverse probability weighting to account for non-representativeness arising from these mechanisms.

For baseline participation, we used previously generated UK Biobank participation probabilities designed to correct for selective baseline participation[1]. These sampling weights were derived using a LASSO regression model predicting participation in baseline assessments (1 = participated; 0 = representative reference sample), based on the same 14 harmonized predictors described previously[1]. For follow-up participation, we generated a second set of weights using the same approach. Specifically, we estimated the probability of participation in prospective follow-up assessments (1 = participated in follow-up; 0 = representative reference sample) using a LASSO regression model with the identical set of predictors.

For the cross-sectional models, baseline sampling weights were incorporated using weighted least squares regression implemented in the R package *survey*[2]. For the longitudinal models applied to the prospective UK Biobank sample, follow-up sampling weights were incorporated into weighted linear mixed-effects models using the R package *svylme*[3].

Supplementary Figure 2 summarizes weighted and unweighted age effects from cross-sectional analyses (Panel **A**) and longitudinal analyses (Panel **B**). Panel **C** displays the absolute difference between the weighted and unweighted estimates, shown separately for the cross-sectional (in blue) and longitudinal models (in red). Overall, weighting had a smaller impact on longitudinal than on cross-sectional estimates ($\text{mean}_{|\beta-\beta_w|}$=0.0018 versus $\text{mean}_{|\beta-\beta_w|}$=0.004, respectively). However, for several traits, the impact of weighting was larger in the longitudinal models than in the cross-sectional models (e.g., BMI, number of cancers), possibly indicating that these traits are more relevant for dropout during follow-up than of initial baseline participation."

R3.3: The phenotype for the change model in (3) is the ratio between the change in phenotype from baseline to the most recent follow-up (ΔP) and the duration of follow-up (FU). Pitfalls in performing GWAS on ratio traits were recently discussed in: https://pubmed.ncbi.nlm.nih.gov/39818621/. In the present case, there are at least two potential pitfalls. First, the model in (3)

$$\frac{\Delta P}{FU} = \alpha + \delta G + \epsilon$$

is numerically identical to the model

$$\Delta P = (FU)\alpha + \delta(FU \cdot G) + FU \cdot \epsilon$$

where only the intercept from W is retained for simplicity. That is, the ratio model implicitly assumes that 1. ΔP increases linearly in time, 2. the effect of G on ΔP interacts with time, and 3. the variance of ΔP is proportional to the follow-up time squared. While these assumptions may or may not be reasonable, they should be acknowledged. Second, when regressing the change ratio on genotype according to model (3), association of G with FU is sufficient to drive an association. Thus, genetic variants that affect a subject's duration of follow-up, for example because they increase the risk of death, will associate with the ratio even if they have no separate effect on ΔP . Please comment on some of these limitations of using the ratio model to detect longitudinal genetic effects.

We agree that the ratio formulation comes with certain assumptions and limitations. In response, we have therefore expanded the Supplement to clarify these assumptions and to discuss the implications of using change ratios in genome-wide analyses.

[Manuscript, Methods]: "The outcome for model 3, P_{Δ_j}, reflecting an individual's annual rate of change in a given phenotype, is calculated as the difference between the most recent follow-up and baseline phenotype ($P_1 - P_0$), divided by follow-up duration (FU): $P_{\Delta_j} = \frac{P_1 - P_0}{FU}$. Further considerations regarding the assumptions and limitations of such ratio traits are provided in the **Supplementary Note**."

[Supplementary Note]: "**Supplementary note on change ratios**

In our two-step genome-wide approach to detect age-varying genetic effects, we used the annualized rate of phenotypic change as the outcome. This was defined as $P_{\Delta_j} =$

$\frac{P_{1j}-P_{0j}}{FU_j}$, where P_{0j} and P_{1j} denote the baseline and most recent follow-up measurement for individual and FU_j is the corresponding follow-up duration. Although this formulation is computationally efficient for large-scale genome-wide analyses, it relies on several assumptions that warrant consideration.

First, because $\frac{P_{1j}-P_{0j}}{FU_j} = \mathrm{W} \cdot \boldsymbol{\alpha} + \delta_{\mathrm{L}} \cdot G_j + \varepsilon_j$ can be rearranged to $P_{1j} - P_{0j} = \boldsymbol{\alpha}(FU_j \cdot \mathrm{W}) + \delta_{\mathrm{L}}(FU_j \cdot G_j) + FU_j \cdot \varepsilon_j$, the ratio formulation implicitly implies that change increases linearly with follow-up time, that the SNP effect on change scales linearly with follow-up duration, and the variance of the change score is proportional to the follow-up time squared - assumptions that are not directly testable within a simple linear regression framework. However, given current sample sizes and realistic interaction effects, detecting age-specific effects other than linearly increasing or decreasing would be severely underpowered in most genome-wide settings. In this context, the assumption of heteroscedastic error may also be reasonable, as many other factors influencing complex traits are likely to operate in an age-dependent manner, consistent with empirical studies showing that the variance of several complex traits increases with age (e.g., BMI[7], SBP[8]).

Second, ratio outcomes can be sensitive to associations with the denominator. If a genetic variant influences follow-up duration, dividing by P_{Δ_j} may induce an association between genotype and the change outcome even in the absence of a direct genetic effect on phenotypic change[7,8]. As a sensitivity analysis, this possibility can be evaluated empirically, for example by conducting genome-wide association analyses with follow-up duration (FU_j) as the outcome.
Given these limitations of difference-score approaches, mixed-effects models are generally preferred for studying age-varying genetic effects[9–11]. Compared to simple change models, mixed-effects models have been shown to better control false positives, yield more precise parameter estimates, and reduce bias in gene-by-age interaction effects in simulation studies[9]. In addition, their flexibility allows explicit evaluation of modeling assumptions, including non-linear age effects as examined here (c.f., Models A4/B4 in the main text). Although computationally more demanding, recent software developments such as GMMAT/MAGEE[11] are now making genome-wide applications of mixed-effects models increasingly feasible.

References

7. Mooldijk, S. S., Labrecque, J. A., Ikram, M. A. & Ikram, M. K. Ratios in regression analyses with causal questions. *American Journal of Epidemiology* **194**, 311–313 (2025).
8. McCaw, Z. R. *et al.* Pitfalls in performing genome-wide association studies on ratio traits. *Human Genetics and Genomics Advances* **6**, (2025).
9. Wiegrebe, S. *et al.* Analyzing longitudinal trait trajectories using GWAS identifies genetic variants for kidney function decline. *Nature communications* **15**, 10061 (2024).
10. Ko, S. *et al.* GWAS of longitudinal trajectories at biobank scale. *The American Journal of Human Genetics* **109**, 433–445 (2022).
11. Zhang, M. *et al.* Robust mixed model association test for gene-environment interactions. *medRxiv* (2025) doi:10.1101/2025.10.01.25336808.

R3.4: Please comment on why model (B2) does not adopt the within-subject age centering adopted for model (A2).

There is not specific reason why the within-subject age centering was not used in the genetic models - in principle, its inclusion should not substantially alter the estimated gene-by-age interaction effects. Hence, to avoid potential confusion and ensure consistency across models, we have now implemented the same within-subject age-centering approach used in Model (A2 and A4) in the longitudinal genetic models (B2) and (B4). We have updated the model equations accordingly and re-estimated the genetic effects. The updated equations are provided below, and the revised results are illustrated in the figure below (original versus updated version), highlighting the minimal impact that this implementation had.

The updated equations:

$$\text{(B2)}\ P_{tj} = \mathbf{W} \cdot \alpha + \beta_{\mathrm{L}} \cdot \underline{\mathrm{age}_{c,tj}} + \gamma_{\mathrm{M}} \cdot G_j + \delta_{\mathrm{L}} \cdot (\underline{\mathrm{age}_{c,tj}} \times G_j) + u_j + \varepsilon_{tj}$$

$$\text{(B4)}\ P_{tj} = \mathbf{W} \cdot \alpha + \beta_1 \cdot \underline{\mathrm{age}_{c,tj}} + \beta_2 \cdot \underline{\mathrm{age}^2_{c,tj}} + \gamma \cdot G_j + \delta_{\mathrm{L}} \cdot (\underline{\mathrm{age}_{c,tj}} \times G_j) + \delta_{\mathrm{Q}} \cdot (\underline{\mathrm{age}^2_{c,tj}} \times G_j) + u_j + \varepsilon_{tj}$$

The updated figure:

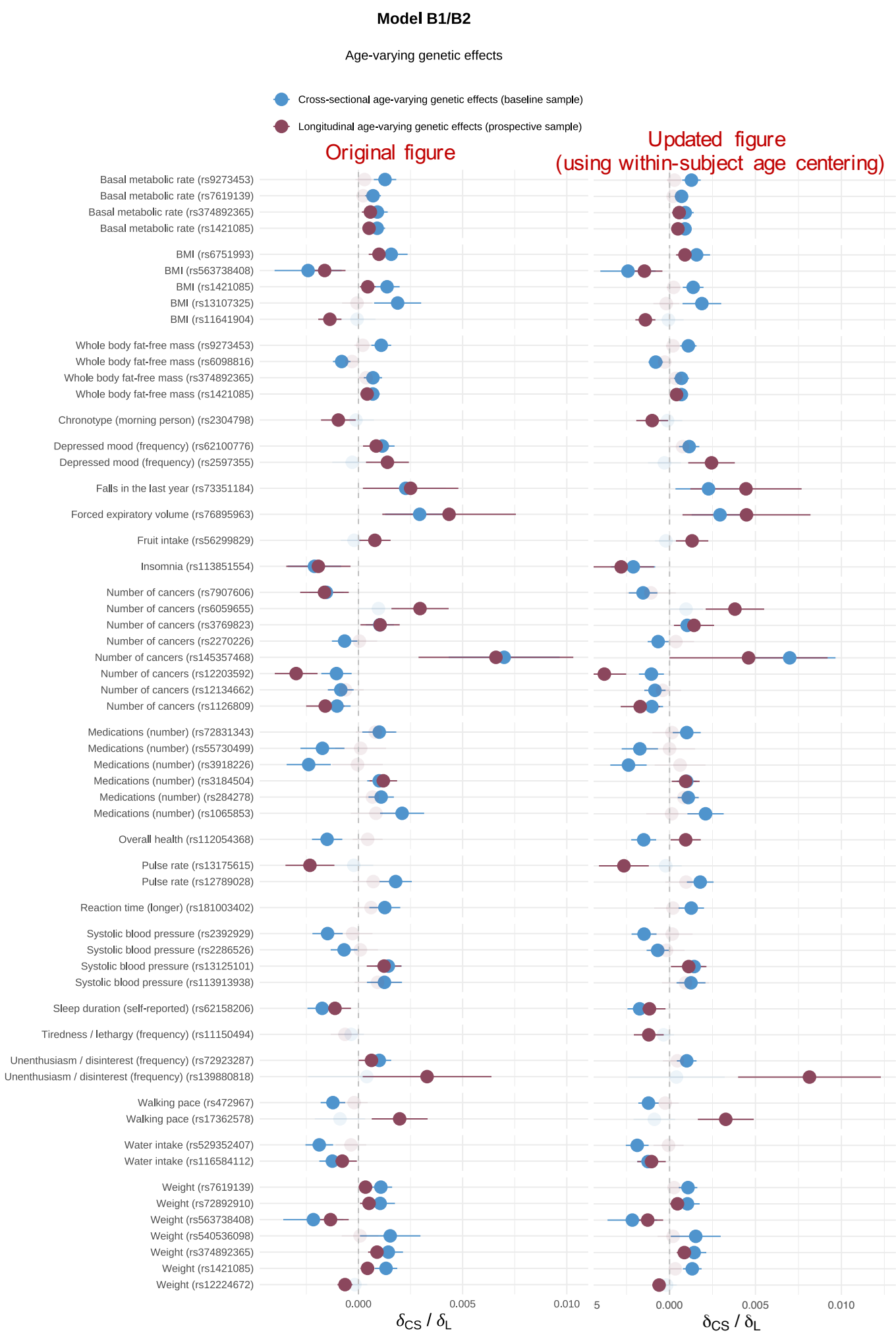

R3.5: When discussing bias, in paragraph 2 of the results and elsewhere, please be careful to specify with respect to what the "bias" is being measured. Statistically, bias is defined as the expected difference between an estimator and the quantity it estimates. That is,

$$Bias(\hat{\theta}) = E(\hat{\theta} - \theta)$$

While it is scientifically interesting that the difference between the longitudinal and cross-sectional effects is almost entirely explained by cohort effects, this is not a consequence of one or the other estimators being statistically biased. Rather, it reflects differences in the target estimands. Taking smoking as an example, there is no contradiction in smoking rates decreasing with age longitudinally yet increasing with age cross-sectionally. The only "bias" here is in conflating a cross-sectional effect with a longitudinal one, which is an error in interpretation not a deficiency of the estimator. This distinction, between statistical bias and target estimand misalignment, needs to be made clear. Overloading "bias" to refer to any difference makes the source of the difference, and consequently the remedy, more difficult to diagnose.

We agree with the reviewer that differences between the cross-sectional and longitudinal estimates do not necessarily reflect bias, as the target estimands can be different. To avoid

confusion, we have now removed the term 'bias' throughout the manuscript where it was used to describe discrepancies between the two designs.

> [Abstract, Manuscript]: "… investigated ~~potential sources of bias underlying any observed discrepancies~~ factors contributing to differences in estimated age-varying genetic effects~~.~~"
>
> [Abstract, Manuscript]: "As neither approach is immune ~~to bias,~~ to design-specific limitations, […]"
>
> [Results, Manuscript]: "~~To investigate potential sources of bias underlying these discrepancies,~~ To better understand the factors contributing to discrepancies between cross-sectional and longitudinal estimates, […]"
>
> [Results, Manuscript]: "[…], its contribution was only small compared to ~~the bias induced by the birth year effect~~ the variation attributable to birth year effects"
>
> [Results, Manuscript]: "[…], representing ~~the principal source of bias in cross-sectional analyses~~ the principal contributor to the observed differences between designs.
>
> [Discussion, Manuscript]: "[…] and investigated ~~key sources of bias that may underlie any observed discrepancies~~ factors that may contribute to any observed discrepancies between designs."
>
> [Discussion, Manuscript]: "Selective participation was identified ~~as the second most relevant source of bias, leading to differences in age-varying genetic effects obtained from the two designs~~ as the second most relevant contributor to differences in age-varying genetic effects obtained from the two designs.

R3.6: Page 13 states: "*Although a significant linear relationship was observed between cross-sectional and longitudinal estimates, the slope was significantly different from one, indicating that cross-sectional estimates were systematically inflated compared to the longitudinal estimates.*" This sentence is again implicitly assuming that the cross-sectional and longitudinal estimates target the same estimand, yet the cross-sectional interaction δ_{CS} in model (B1) and the longitudinal δ_{L} in model (B2) are separate and distinct parameters. Specifically, (B1) estimates the parameter

$$\frac{\partial}{\partial Age_{0j}}\{E(P_{0j}|W, Age_{0j}, G_j = 1) - E(P_{0j}|W, Age_{0j}, G_j = 0)\}.$$

Meanwhile, (B2) estimates the parameter

$$\frac{\partial}{\partial Age_{tj}}\{E(P_{tj}|W, Age_{tj}, G_j = 1) - E(P_{tj}|W, Age_{tj}, G_j = 0)\}.$$

Heuristically, (B1) is asking whether the effect of genotype varies with age across subjects at a specific time point, while (B2) is asking whether the effect of genotype varies with age within subjects. The answers to these two questions need not be the same. It is misleading to

say the cross-sectional estimates are "systematically inflated" compared to the longitudinal estimates; they are not measuring the same quantity.

We agree with the reviewer's comment have revised that sentence in the results as follows:

> [Results, Manuscript]: "Although a significant linear relationship was observed between cross-sectional and longitudinal estimates, the slope differed significantly from one, indicating that cross-sectional effect sizes were s~~ystematically inflated compared to the longitudinal estimates~~ systematically larger in magnitude than longitudinal estimates."

R3.6: Minor comments

A. On the bottom of the first page of the introduction, the text should say "due to differences".
B. In the following sentence, consider replacing "only one single repeat assessment" by "only one assessment". Later, the sentence should say "at least three time points of data", or better yet "at least three measurements". ("*Excluded were all phenotypes with only one single repeat assessment available, as parts of our modelling approach relied on the use of a subset of UKB participants with at least three-time point data to explore possible non-linear age effects (c.f., below).")*
C. C. At the bottom of the second paragraph under Methods, the text should say "Each phenotype … was standardized prior to inclusion". As the phenotypes are also being centered, this is not simply scaling.

The three issues raised by the reviewer have now been corrected.

Remarks on code availability: I was not able to run the code, though in general it appears to be well-documented. I would suggest the authors apply tidyverse styling to their R code.

The repository links have now been corrected. Regarding "tidyverse styling," our pipeline currently uses a mix of base R and domain-specific packages (e.g., for mixed modeling, file handling, and table generation). Tidyverse components were used whenever they proved useful (e.g., ggplot2, and selected dplyr/tidyr utilities).

Design and model choices shape inference of age-varying genetic effects on complex traits

This manuscript addresses the distinction between cross-sectional and longitudinal age-varying genetic effects, an important topic where confusion abounds. The approach is rigorous, the exposition is clear, and the conclusion that significant cohort effects exist for many of the traits studied is scientifically significant. My main suggestion relates to the manuscript's current framing. In particular, the current exposition implicitly assumes that the cross-sectional and longitudinal models target the same estimand. In general, they do not. To avoid perpetuating this misconception, please state clearly and early on that these analyses target different estimands and, consequently, their estimates need not coincide (in general). Only under strong assumptions, which the manuscript should make explicit (e.g., no cohort- or period-by-genotype effect modification), are estimates from the two models expected to agree. Absent this context, describing the cross-sectional estimates as "biased" relative to the longitudinal estimates conflates *estimator bias* with *estimand mismatch*.

Main

1. In the introduction, consider defining and contrasting cohort and age effects, and explaining why cohort effects can be mistaken for age effects without careful modeling.
2. Models (A5) and (B5) use inverse probability weighting to account for baseline participation. Was an analogous inverse-probability-of-censoring weighting analysis considered to address potentially informative dropout during follow-up? Relatedly, please estimate, if possible, the proportion of subjects lost to follow-up due to death, and please comment on how attrition due to death impacts the analysis.
3. The phenotype for the change model in (3) is the ratio between the change in phenotype from baseline to the most recent follow-up (ΔP) and the duration of follow-up (FU). Pitfalls in performing GWAS on ratio traits were recently discussed in: https://pubmed.ncbi.nlm.nih.gov/39818621/. In the present case, there are at least two potential pitfalls. First, the model in (3)
$$\frac{\Delta P}{FU} = \alpha + \delta G + \epsilon$$
is numerically identical to the model
$$\Delta P = (FU)\alpha + \delta(FU \cdot G) + FU \cdot \epsilon$$
where only the intercept from W is retained for simplicity. That is, the ratio model implicitly assumes that 1. ΔP increases linearly in time, 2. the effect of G on ΔP interacts with time, and 3. the variance of ΔP is proportional to the follow-up time squared. While these assumptions may or may not be reasonable, they should be acknowledged. Second, when regressing the change ratio on genotype according to model (3), association of G with FU is sufficient to drive an association. Thus, genetic variants that

affect a subject's duration of follow-up, for example because they increase the risk of death, will associate with the ratio even if they have no separate effect on ΔP. Please comment on some of these limitations of using the ratio model to detect longitudinal genetic effects.

4. Please comment on why model (B2) does not adopt the within-subject age centering adopted for model (A2).
5. When discussing bias, in paragraph 2 of the results and elsewhere, please be careful to specify with respect to what the “bias” is being measured. Statistically, bias is defined as the expected difference between an estimator and the quantity it estimates. That is,

$$Bias(\hat{\theta}) = E(\hat{\theta} - \theta)$$

While it is scientifically interesting that the difference between the longitudinal and cross-sectional effects is almost entirely explained by cohort effects, this is not a consequence of one or the other estimators being statistically biased. Rather, it reflects differences in the target estimands. Taking smoking as an example, there is no contradiction in smoking rates decreasing with age longitudinally yet increasing with age cross-sectionally. The only “bias” here is in conflating a cross-sectional effect with a longitudinal one, which is an error in interpretation not a deficiency of the estimator. This distinction, between statistical bias and target estimand misalignment, needs to be made clear. Overloading “bias” to refer to any difference makes the source of the difference, and consequently the remedy, more difficult to diagnose.
6. Page 13 states:

Although a significant linear relationship was observed between cross-sectional and longitudinal estimates, the slope was significantly different from one, indicating that cross-sectional estimates were systematically inflated compared to the longitudinal estimates.

This sentence is again implicitly assuming that the cross-sectional and longitudinal estimates target the same estimand, yet the cross-sectional interaction δ_{CS} in model (B1) and the longitudinal δ_{L} in model (B2) are separate and distinct parameters. Specifically, (B1) estimates the parameter

$$\frac{\partial}{\partial Age_{0j}}\{E(P_{0j}|W, Age_{0j}, G_j = 1) - E(P_{0j}|W, Age_{0j}, G_j = 0)\}.$$

Meanwhile, (B2) estimates the parameter

$$\frac{\partial}{\partial Age_{tj}}\{E(P_{tj}|W, Age_{tj}, G_j = 1) - E(P_{tj}|W, Age_{tj}, G_j = 0)\}.$$

Heuristically, (B1) is asking whether the effect of genotype varies with age *across* subjects at a specific time point, while (B2) is asking whether the effect of genotype varies with age *within* subjects. The answers to these two questions need not be the

same. It is misleading to say the cross-sectional estimates are “systematically inflated” compared to the longitudinal estimates; they are not measuring the same quantity.

Minor

1. On the bottom of the first page of the introduction, the text should say “due **to** differences”.
2. In the following sentence, consider replacing “only one single repeat assessment” by “only one assessment”. Later, the sentence should say “at least three time **points of** data”, or better yet “at least three measurements”.

   Excluded were all phenotypes with only one single repeat assessment available, as parts of our modelling approach relied on the use of a subset of UKB participants with at least three-time point data to explore possible non-linear age effects (c.f., below).

3. At the bottom of the second paragraph under Methods, the text should say “Each phenotype … was **standardized** prior to inclusion”. As the phenotypes are also being centered, this is not simply scaling.
